# Challenges and Opportunities of Light-Emitting Diode (LED) as Key to Modulate Antioxidant Compounds in Plants. A Review

**DOI:** 10.3390/antiox10010042

**Published:** 2020-12-31

**Authors:** Martina Loi, Alessandra Villani, Francesco Paciolla, Giuseppina Mulè, Costantino Paciolla

**Affiliations:** 1 Institute of Sciences of Food Production, National Research Council, Via Amendola 122/O, 70126 Bari, Italy; alessandra.villani@ispa.cnr.it (A.V.); giuseppina.mule@ispa.cnr.it (G.M.); 2 Department of Biology, University of Bari Aldo Moro, Via E. Orabona 4, 70125 Bari, Italy; costantino.paciolla@uniba.it; 3 Automation Engineering, Polytechnic of Bari, Via E. Orabona 4, 70125 Bari, Italy; paciollafrancesco0411@gmail.com

**Keywords:** food quality, food safety, light-emitting diode (LED), plant antioxidants, polyphenols, postharvest, vitamin C

## Abstract

Plant antioxidants are important compounds involved in plant defense, signaling, growth, and development. The quantity and quality of such compounds is genetically driven; nonetheless, light is one of the factors that strongly influence their synthesis and accumulation in plant tissues. Indeed, light quality affects the fitness of the plant, modulating its antioxidative profile, a key element to counteract the biotic and abiotic stresses. With this regard, light-emitting diodes (LEDs) are emerging as a powerful technology which allows the selection of specific wavelengths and intensities, and therefore the targeted accumulation of plant antioxidant compounds. Despite the unique advantages of such technology, LED application in the horticultural field is still at its early days and several aspects still need to be investigated. This review focused on the most recent outcomes of LED application to modulate the antioxidant compounds of plants, with particular regard to vitamin C, phenols, chlorophyll, carotenoids, and glucosinolates. Additionally, future challenges and opportunities in the use of LED technology in the growth and postharvest storage of fruits and vegetables were also addressed to give a comprehensive overview of the future applications and trends of research.

## 1. Introduction

Plant antioxidants include a wide variety of compounds, which are responsible for essential plant functions, including signaling, defense, oxidative damage prevention, and free-radical scavenging [1]. In addition, some antioxidants are responsible for the color, aroma, and taste of fruits, vegetables, and processed products. Thus, they determine the quality and shelf life of a food, consumers’ appreciation, and their economic value [2]. Fruits, vegetables, herbs, and spices are rich in antioxidant compounds, and their consumption in the diet is encouraged, owing to their antioxidant and anti-inflammatory properties, the positive effects on blood pressure, lipids, insulin resistance, and cardiovascular health [3]. Several meta-analyses reported evidence of an inverse correlation between antioxidants in the diet and all-cause mortality [4] and acute coronary syndrome [3], even among high-risk subjects [5], whilst the consumption of specific polyphenols from olives and cocoa was recognized as beneficial to prevent cardiovascular disease [6,7].

Polyphenols, photosynthetic pigments, glutathione, vitamin C (L-ascorbic acid, Vit C) and other vitamins, and antioxidant enzyme systems, such as generic peroxidases, polyphenol oxidases, ascorbate peroxidase (APX), glutathione peroxidase, glutathione reductase (GR), monodehydroascorbate reductase (MDHAR), dehydroascorbate reductase (DHAR), thioredoxins, peroxiredoxin, superoxide dismutase, and catalase are among the most important components involved in the modulation of the redox status of the cells and in the response to pathogens and adverse environmental conditions. Several factors are involved in the regulation of the synthesis, turnover, and degradation of such compounds, including both biotic and abiotic stresses, such as light [8,9,10]. Development, growth, and physiology of the plant depend on the availability of light [11,12]. Light duration (photoperiod), intensity, quality (wavelength), and direction play key roles in plant studies [13].

Through photosynthetic fixation, plants are able to transform the solar energy into reducing equivalents, and CO_2_ into sugars. However, light is an essential factor driving several biochemical pathways for plant growth and development. In particular, the synthesis and accumulation of antioxidants such as Vit C, phenolic acids, carotenoids, flavonoids, anthocyanins, and α-tocopherol in leafy tissues and fruits in many horticultural and herbal crops are driven by the photoperiod, intensity, and quality [14,15,16]. Light intensity and wavelength vary during the daytime and by season, latitude, and climate and are perceived differently by cells according to the position of the leaf within a canopy and of the cell within a leaf [17].

Light quality and intensity are perceived by plants through different types of photoreceptors, namely, phytochrome (PHY), cryptochrome (CRY), phototropin (PHOT), flavin binding Kelch domain F box protein (FKF1), zeitlupe (ZTL), LOV Kelch protein2 (LKP2), and UV-B resistance locus 8 (UVR8). According to the receptor type, they can be sensitive to both low and high irradiance levels, as well as to specific light wavelengths [18]. Due to such implications, artificial light supplementation has been widely applied in horticulture on economically relevant crops to compensate for short photoperiods, to support photosynthesis, to control plant flowering and pests, and to improve plant nutritional quality [19].

The earliest report of the use of artificial lighting systems dates back to 1868, when the Russian botanist Andrei Famintsyn applied artificial light in plant and microalgae research [20]. Since then, different lighting systems have been used, including fluorescent, high-pressure mercury, high-pressure sodium (HPS), metal-halide lamps, and light-emitting diode (LED).

At present times, LED technology has gained a massive popularity for its ability to produce specific spectra. In fact, compared to other artificial light sources, monochromatic LEDs show unique spectra outputs in terms of wavelengths, along with an equivalent luminous efficacy, lower operational cost, lack of radiant heat, and longer lifespan. Due to these unique advantages, LEDs are now used in controlled environments, e.g., growth chambers, greenhouses, and vertical farming, as well as in the postharvest storage of many vegetables to support plant growth and to specifically stimulate the synthesis of bioactive compounds [21].

LEDs allow studying the light-induced processes and, in particular, the light-dependent (or induced) physio-morphological events that could bring, for instance, increased production of primary and secondary metabolites, or the biosynthesis of novel molecules with health-promoting properties.

However, regulating the production of specific antioxidant compounds by means of LEDs is a complex task. The metabolic pathways are often redundant and regulated at different levels, according to the plant species, tissues, the spectral combination, photoperiods, and irradiances [2,22]. Therefore, this review aims to elucidate LEDs effects on plants metabolism, with particular regard to the content of antioxidant compounds, such as Vit C, phenols, chlorophylls, carotenoids, and glucosinolates.

An overview of the most recent findings in the horticultural field is provided, and the challenges and perspectives of LEDs application at all levels of the supply chain are critically discussed.

## 2. LED Technology

**Figure 1:**
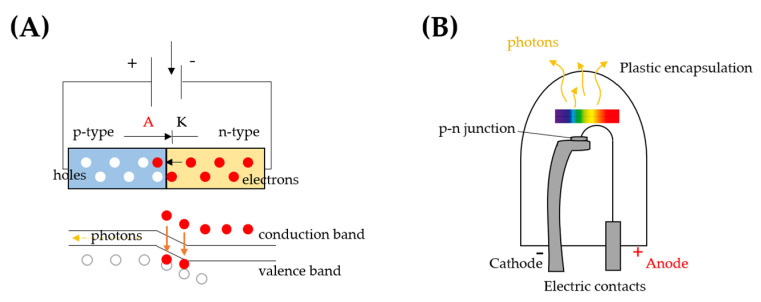
(**A**) Working principle and (**B**) structure of a light-emitting diode (LED).

An LED is a solid-state semiconductor diode, allowing unidirectional current flow from the anode to the cathode within a specific voltage range. The diode is composed of two differently doped materials that jointly form a p-n heterojunction, where the p-side contains excess positive charge (holes), while the n-side contains excess negative charge (electrons). When a positive forward voltage is applied, the current flows from the p-side to the n-side and electrons are forced to move from the n-side toward the p-side, whilst holes make the reverse path. As an electron crosses the depletion layer, near the junction, it recombines with a hole and falls from the conduction band in a lower energy level, the valence band, and releases energy in the form of a photon (Figure 1A). The effect is a form of electroluminescence and incoherent narrow-spectrum light is emitted.

In Figure 1B, the most important parts of an LED are shown. The cathode is connected to the n-side of the junction, while the p-side is connected to the anode with a connecting wire.

The emitted light wavelength, so its color, depends on the band gap energy of the junction’s materials [23,24]. The band gap represents the minimum energy difference between the top of the valence band and the bottom of the conduction band; it depends on the dopants used in the p- and n-sides. An LED is usually made by direct band gap semiconductors, so the top of the valence band and the bottom of the conduction band occur simultaneously; in this way, the probability that an electron spontaneously recombines with a hole is high. 

The electron–hole recombination rate r is

(1) r=Bnp 

where B is a constant and depends on the material properties of the semiconductors being used, and n and p are the concentrations of electrons and holes in atoms/cm^3^, respectively.

If the transitions take place in a direct bandgap semiconductor, the emission wavelength λ is

(2) $$\lambda =hc/E_{g}$$

where h is Planck’s constant, c is the speed of light and $E_{g}$ is the energy band gap.

In order to obtain visible radiation, energy gaps have to be greater than about 2 eV; unfortunately, materials with such a large gap tend to have high resistivities even when doped.

**Figure 2:**
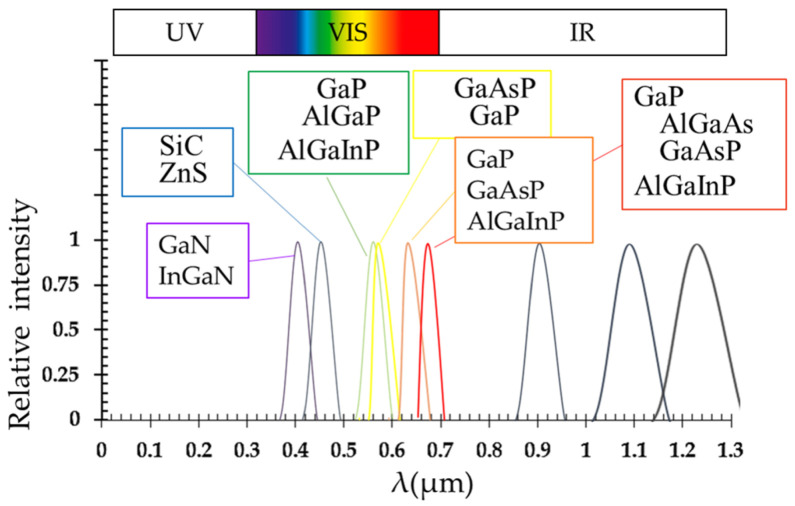
Spectral distribution emitted by an LED fabricated from various semiconductors (adapted from [25]).

The most common commercial LEDs require a forward operating voltage approximately between 1.6 and 4 V with a forward current rating from 10 to 30 mA, with 12 to 20 mA being the most common range. The spectral densities emitted by LEDs fabricated from various semiconductors and their characteristics are shown (Table 1; Figure 2). The emission spectrum becomes narrower in terms of wavelength as the emission energy moves from the infrared to the ultraviolet ends of the spectrum [25].

There are different performance parameters to compare LEDs. The most important one is the external quantum efficiency $\eta_{EQE}$ (EQE) which quantifies the conversion efficiency of electrical energy into external optical energy:

(3) $$\eta_{EQE}=\eta_{IQE}\times \eta_{EXT}$$

where $\eta_{IQE}$ is the internal quantum efficiency (IQE), which is the ratio between the number of emitted photons from the active region per second and the injected electrons per second, and $\eta_{EXT}$ is the extraction efficiency which represents the ratio between the number of photons generated that escape from the device per second and the number of emitted photons from the active region per second [24].

**Figure 3:**
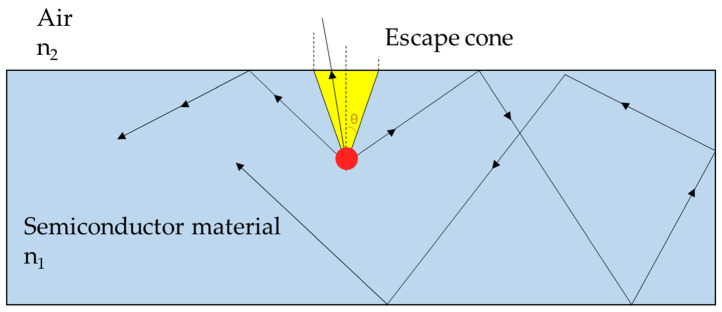
Light escape cone (figure adapted from [27]).

The IQE of today’s best LEDs is at least 75% and may even be approaching 80%. To make further improvements, nonradiative recombination centers need to be eliminated and a shift to semipolar crystallographic LEDs is needed [26]. Although the IQE is high, the EQE is much lower; the radiation at the semiconductor/air interface will escape only when the incident rays form an angle with the normal to the interface within the cone of critical angle $\theta_{c}$, whilst other rays get reflected via total internal reflection (TIR) in the semiconductor (Figure 3). The critical angle is calculated as

(4) $$\theta_{c}=sin^{-1}\left(n_{2}/n_{1}\right)\;\mathrm{with}\;n_{2}<n_{1}$$

Unfortunately, most of the LED materials are III-V compounds of the periodic table and have high refractive indices giving rise to small critical angles, hence the escape cone is very small. For instance, gallium arsenide-based materials, common for red LEDs, have a very high refractive index (*n* = 3.4) and the escape cone has $\theta_{c}$ = 17.1° [24], meaning that only about 2% of the light can escape. One of the most used techniques to widen the escape cone and increase the EQE is to surround the junction in a transparent plastic encapsulation ($n_{2}=1.5$).

## 3. Plant Photoreceptors

Plants utilize light to produce energy by the photosynthetic process and to obtain information on the habitat where they live. The perception of the light stimulus is made possible by the photoreceptors, specialized proteins able to perceive specific wavelengths of spectra light, from 250 to 750 nm.

**Figure 4:**
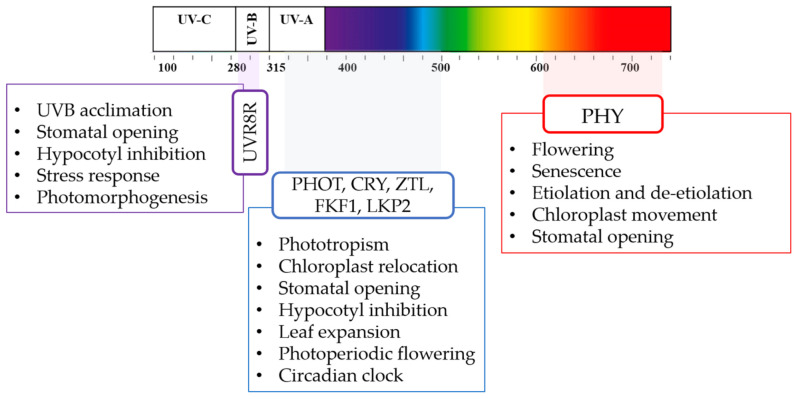
Light spectra, plant photoreceptors, and main light-driven physiologic plant response. Ultraviolet radiation A (UV-A), ultraviolet radiation B (UV-B), ultraviolet radiation C (UV-C), phytochrome (PHY), cryptochrome (CRY), phototropin (PHOT), flavin binding Kelch domain F box protein (FKF1), zeitlupe (ZTL), LOV Kelch protein2 (LKP2), and UV-B resistance locus 8 (UVR8).

In the Brassicaceae *Arabidopsis thaliana*, a model plant organism, four main classes of photoreceptors were described: PHYs, which perceive red light (RL, absorption peak of ~660 nm) and far red light (FR, absorption peak of ~730 nm) [28], CRYs [29], PHOTs [30], and three recognized LOV/F-box/Kelch-repeat proteins, namely, ZTL, FKF1, and LKP2 [31,32], which absorb ultraviolet A radiation (UV-A, ~315–400 nm) or blue light (BL) (~400–500 nm, with a peak of absorption ~470 nm). In addition, one UV-B (~280–315 nm) photoreceptor, called UVR8, was identified [33,34] (Figure 4). So far, in *Arabidopsis*, five different phytochromes have been identified, namely, PHY A, B, C, D, and E. PHY B perceives RL (660 nm) that converts the physiological inactive form of the Pr phytochrome into a physiological active form of the Pfr phytochrome and triggers different physiological processes. PHY B controls physiological responses known as escape from shade, in which transcriptional factors called phytochrome interacting factors (PIFs) regulate the expression of the genes involved, according to the R/FR ratio. In addition, PHYs regulate the flowering, senescence, etiolation, and de-etiolation. BL is mainly perceived by three receptor families: CRYs, PHOTs, and ZTLs [35]. By these photoreceptors, BL modulates various processes, such as phototropism, chloroplast relocation, stomatal opening, rapid inhibition of hypocotyl elongation, and leaf expansion [36]. In *Arabidopsis*, there are three different cryptochromes: CRY1, CRY2, and CRY3. CRY1 and CRY2 proteins are involved in de-etiolation, which is BL intensity-dependent [37].

Together with phytochromes, CRYs are involved in the escape from shade (canopy shade) [38], whilst all blue photoreceptors are involved in the circadian rhythm. Many metabolic activities show a circadian rhythm and therefore they are regulated by endogenous factors, namely, oscillators, that supervise the rhythm also in absence of external stimuli [39]. For instance, the flowering is influenced by the photoperiod and the hours of light are fundamental for the plant to perceive the seasonal variations and to flower in a favorable annual period. 

There are two PHOTs in *Arabidopsis,* namely, PHOT1 and PHOT2. Both regulate the phototropism in elevated BL intensity (1–100 µmol m^−2^·s^−1^), while PHOT1 also regulates in low BL intensity (0.01–1 µmol m^−2^·s^−1^) [40]. By modulating the stomatal opening, the phototropins also regulate CO_2_ uptake and transpiration (PHOT1 and PHOT2) and modulate chloroplast movements (PHOT2). FKF1 is essential for photoperiodic-specific light signaling in *Arabidopsis* [41] and F-box proteins FKF1 and LKP2 act synergistically with ZTL to control *Arabidopsis* clock progression [42]. UVR8 mediates UV-B photomorphogenic responses, securing plant acclimation and thus promoting plant survival and photoprotection in sunlight [33]. Generally, all photoreceptors perceive, interpret, and transduce light signals via distinct intracellular signaling pathways, by means of which the photoresponsive nuclear gene expression is regulated, finally leading to adaptive changes at the cell and whole organism levels [43].

Interestingly, as in nature, the lighting conditions are very dynamic and changing, and plants are able to perceive RL, BL, and FR light at the same time. In this way, plants are able to create a balance between growth, yield, and the accumulation of compounds with antioxidant activity. Studying the interplays of the photoreceptors-mediated signaling transduction pathways could bring researchers to a better understanding of the molecular mechanisms of such interactions and of the processes they regulate. A useful experimental approach for this study could exploit LEDs, which enable the use of selected wavelengths and intensities. From a practical point of view, the biggest challenge is to design an LED protocol which can be modulated to follow the plant through its phenological stages and provide stimuli to the growth, the development of plant biomass, or the accumulation of antioxidant compounds when needed. The recent advancements of LED technologies provide abundant opportunities to study those various plant light responses to monochromatic or specific light combinations, especially with regard to the antioxidant and phytonutrient compounds of plants [44].

## 4. LED Effects on the Antioxidants and Phytonutrients of Plants

### 4.1. Vitamin C

Vit C is an important antioxidant compound which is ubiquitous in the plant kingdom. It is a natural water-soluble vitamin with a low molecular weight (176.12 Da) and possesses numerous functions in plants [45,46,47,48]. In plants, Vit C is involved in growth and development processes and, in particular, in the synthesis of plant hormones, in cell division, cell elongation, and cell differentiation, in programmed cell death, in photosynthesis, and in iron uptake, as well as in defense response against biotic and abiotic stresses [45,48,49]. The major pathway of ascorbate synthesis in higher plants is the L-galactose pathway (Figure S1) [48,50]. In humans, Vit C is a potent reducing and antioxidant agent that is involved in the formation of collagen in fibrous tissue, teeth, bones, connective tissue, skin, and capillaries. Furthermore, it is involved in detoxifying reactions and can prevent bacterial infections [51]. Vit C cannot be produced or stored by humans; therefore, it must be assumed with vegetables and fruits in the diet. The best dietary sources include green pepper, strawberries, oranges, and blackcurrants, which contain approximatively 60–100 mg/100 g of fresh weight [52]. Light can control Vit C accumulation through different types of regulation [48]. Vit C synthesis is light-dependent, as reported in *Phaseolus vulgaris* leaves [53], oat (*Avena sativa* L., cv. Roga) leaves [54], postharvest broccoli (*Brassica oleracea* L. var. *italica*) [55,56], green asparagus (*Asparagus officinalis* L. var. UC157) [57], soybean (*Glicine max* L. Merr), mung bean (*Vigna radiata* L. Wilczek), radish (*Raphanus sativus* L.), and pumpkin (*Cucurbita moschata* Duch) sprouts [58]. Its synthesis depends on the intensity and quality of light, as well as exposure time. Consequently, diurnal variations were observed [59,60]. A strong daily change in transcript levels of enzymes of the Vit C biosynthetic pathway was reported in tomato leaves (11 of the 12 studied genes showed significant changes in their expression pattern). Among these genes, a diurnal variation in transcript levels of *VTC2* (coding for GDP-L-galactose phosphorylase) and *GME1* (coding for GDP-D-mannose 3′,5′-epimerase) was correlated with the leaf Vit C content [61]. On the other hand, darkness promotes Vit C catabolism [62]. Increased Vit C accumulation was found in plants grown in open fields with respect to those grown in greenhouses [61] and in sun-illuminated leaves [63]. The constitutive photomorphogenesis 9 (COP9) signalosome (CSN) is one of the main factors involved in Vit C synthesis: it is a photomorphogenic complex of the ubiquitin–proteasome pathway, involved in the degradation of the enzymes involved in ascorbate synthesis in the dark through regulation of E3 ligase activity [64]. As the synthesis of Vit C and the photosynthetic process are strictly correlated, light could influence the Vit C levels by stimulating the photosynthetic process. In fact, soluble carbohydrates are the main photosynthetic products and, at the same time, the precursors of Vit C [65,66]. In contrast, Ntagkas and coworkers [67] showed that in tomato fruits, light induced Vit C accumulation independently from carbohydrate availability, suggesting that other mechanisms may have a significant impact on Vit C synthesis.

Various roles in the regulation of the photosynthetic process have been attributed to Vit C, particularly in the acclimation to high light, as demonstrated in ascorbate-deficient mutants of *Arabidopsis* [68] and in transgenic tobacco lines (*Nicotiana tabacum*) [69] when grown under high irradiance levels. The content of Vit C depends upon the activity of the enzymes involved in its biosynthesis and oxidation, and both have been reported to be influenced by LEDs [70,71]. BL and RL were the most used LEDs to increase Vit C content in different fruits and vegetables; fewer studies have focused on other LED types, such as green (GL) or yellow (YL) LEDs.

BL treatment effectively increased the Vit C content in the juice sacs of three citrus varieties: satsuma mandarin (*Citrus unshiu* Marc.), Valencia orange (*C. sinensis* Osbeck), and Lisbon lemon (*C. limon* Burm.f.). In addition, continuous irradiation was more effective than pulsed irradiation. Particularly, Vit C accumulation by BL treatment was highly regulated at the transcription level with the up-regulation of Vit C biosynthetic genes (*CitVTC1, CitVTC2, CitVTC4,* and *CitGLDH)*, regeneration genes (*CitMDAR1, CitMDAR2,* and *CitDHAR*), and two glutathione-producing genes (*CitGR* and *CitchGR*) [72].

Postharvest irradiation of RL was optimal for delaying the senescence and increasing Vit C content in leaves of pack choi (*Brassica rapa* ssp. *Chinensis*). Gene expression analysis revealed that the *VTC2* biosynthetic gene was 2.54- and 1.32-fold up-regulated after two and four days of RL treatment, respectively, and was down-regulated after dark and FR light treatments; the *VTC4* gene also exhibited a relatively higher expression after RL treatment. The authors concluded that RL might also promote Vit C biosynthesis, at least at the early stage of storage, while BL treatment had little effect on Vit C content [73]. Gene expression analysis confirmed that RL treatment promoted the expression of *BraVTC2*, the putative homologous gene of *AtVTC2* encoding a key Vit C biosynthetic enzyme in *Arabidopsis* [74]. Conversely, in another brassica species, namely, broccoli (*Brassica oleracea* L. var. *italica*), RL effectively suppressed the decrease in Vit C after harvest, whereas BL had no effect on the metabolism of Vit C. The up-regulation of two Vit C biosynthetic genes (*BO-VTC2* and *BO-GLDH*) and two Vit C regeneration genes (*BO-MDAR1* and *BO-MDAR2*) contributed to the higher Vit C content in the modified white light (WL) treatment on the first and second days after harvest [55]. BL proved to be more efficient than RL in strawberry (*Fragaria ananassa* Duch. Cv. Fengguang), which showed higher Vit C content, ascorbate peroxidase activity, and other antioxidant enzymes activities when stored under BL [75].

Changes in levels of Vit C in many vegetables have been observed as a response to the use of single as well as combined spectral LEDs. Supplementing GL with RL plus BL reduced the Vit C content of green leaf lettuce (*Lactuca sativa* L. cv. Lvdie) by 44% compared to RL plus BL. In purple leaf lettuce (*Lactuca sativa* L. cv. Ziya) grown under WL plus RL, no significant differences were observed in Vit C content compared with WL [76]. In lettuce plants under continuous light for 12 days, the ratio 25RL/75BL increased the Vit C level mainly by promoting Vit C regeneration rather than its biosynthesis. In this case, only the expression of regeneration genes (*APX, MDHAR, DHAR,* and *GR*) was significantly increased [77]. The concentration of Vit C was greater in non-heading Chinese cabbage (*Brassica campestris* L) seedlings under BL plus RL, followed by those grown under BL, and fluorescent lamps [78]. This is consistent with reports by [79] and [80]. A significant increase in Vit C concentration was found in sprouted seeds of lentil (*Lens esculenta* Moenh.) and wheat (*Triticum aestivum* L.) under LED spectra supplemented with GL and in radish (*Raphanus sativus* L.) sprouts grown with supplemental amber (595 nm) light [81].

Furthermore, the irradiance level has been shown to have a great impact on Vit C content. Detached tomato (*Solanum lycopersicum)* fruits treated with different irradiance levels in a climate-controlled environment contained 32% more Vit C compared to the fruits kept at lower irradiances or in darkness [82].

On the other hand, GL and YL effects have been scarcely investigated. Vit C content and shoot biomass have been examined in crisphead lettuce (*Lactuca sativa* L. var. *capitata*) by using GL, which has a lower light absorptance but greater light transmittance in leaves. The Vit C content was high in crisphead lettuce, especially under GL irradiation because of the deeper light transmittance [83]. When apple peel, tomato, and red bell pepper fruit were irradiated with YL, there was no significant increase in Vit C content compared to the control samples stored in the dark [84].

As shown in Table 2, few studies evaluated the effect of different wavelengths. The effects of different LEDs were investigated with the aim of maintaining freshness and nutrition in cabbage (*Brassica rapa*) stored at 4–5 °C for 18 days. Vit C content was highest for BL, followed by WL, GL, RL, and a non-irradiated control group. Conversely, Loi and coworkers [56] found that GL was the most efficient LED to improve the Vit C profile in broccoli (*Brassica oleracea* var. *italica*) during postharvest storage, followed by RL and YL [56]. Although these results are not fully comparable, they may suggest that LED colors have different effects also within the same species [85]. Further details about the Vit C biosynthetic pathway and light gene regulation are shown in Figure S1.

### 4.2. Polyphenols

Polyphenols are a widespread group of phytochemicals characterized by the presence of one or more hydroxylated aromatic rings; they are generally subgrouped into phenolic acids, stilbenes, flavonoids, lignans, and ellagic acids [86]. Even though their chemical structure may differ significantly, their synthesis originates from a common intermediate, phenylalanine, obtained through the shikimate pathway (Figure S2). The key enzymes involved in this pathway are the 3-deoxy-D-arabino-heptulosonate 7-phosphate, the DAHP synthase (DS), and the chorismate mutase (CM). Phenylalanine can be converted into phenolic compounds in the phenylpropanoid pathway through the action of another key enzyme, the phenylalanine ammonia lyase (PAL). Those enzymes can be regulated at different levels, including by reactive oxygen species (ROS) deriving from excess light [87] and the activity of light-responsive transcription factors [88].

Polyphenols participate in the plant’s defense system against heavy metals, salinity, drought, extreme temperature, pesticides, and UV radiations. In particular, exposure to UV-B radiation stimulates the synthesis and accumulation of polyphenols in the epidermal tissues, where they absorb both visible (anthocyanins) and UV light (anthocyanins and flavonoids) to protect the mesophyll from photooxidation [89,90]. Fruits are among the polyphenol-richest plant tissues and may contain as much as 200–300 mg of polyphenols per 100 g of fresh weight. Besides providing defense from oxidation, they contribute to the aroma and color, together with the high antioxidant activity, of such products [86].

LEDs have been used to increase polyphenol content, with particular regard to flavonoids. BL, RL, and FR are among the most studied monochromatic LEDs and have shown to be able to increase the total phenol content (TPC) and total flavonoid content (TFC) in different commodities, when applied during germination of sprouts [91,92], plant growth [93,94,95], or in the postharvest storage [56,96]. More specifically, BL has often been reported to increase the content of simple phenols, such as chlorogenic, gallic, ferulic, *p*-coumaric, caffeic, hydroxybenzoic, and benzoic acids. [95,96,97]. Similarly, BL was reported to increase the flavonoids contents, e.g., chalcone, naringenin, quercetin [95], kaempferol, rutin, (+)-catechin, (−)-epicatechin, cyanidin glucosides, and resveratrol [96,97,98,99,100], but also to reduce the content of rutin [99], quercetin, and other flavonoids [95]. During postharvest, RL and BL maintained the levels of different flavonoids, e.g., diosmin, hesperidin, didymin, neoeriocitrin, and narirutin [96]. RL and YL were reported to stimulate the accumulation of simple phenols, such as of *p*-hydroxybenzoic acid, caffeic acid, *p*-coumaric acid, ferulic acid, and flavonoids, such as rutin and resveratrol, though to a lesser extent than BL [98].

Despite the majority of the studies being only focused on the measurement of the TPC, TFC, or of specific phenolic compounds, few of them also reported that these increments after WL, BL, and RL treatments were due to the increased activity of the key enzymes of the shikimate and phenylpropanoid pathways, such as PAL [101,102], chalcone synthase (CHS), chalcone isomerase (CHI), or in flavonoid and anthocyanin synthesis pathways, such as flavonol synthase (FLS), leucoanthocyanidin dioxygenase (LDOX), dihydroflavonol 4-reductase (DFR), and stilbene synthase (STS) (for more details, see Figure S2) [103,104,105]. The synthesis of phenolic compounds is regulated by structural genes, transcriptional activators, and repressors, the latter two acting on putative light regulatory units in gene promoters [106]. In addition, the synthesis of phenolic compounds was shown to be supported by an increased production of phenylalanine opposed to tryptophan. This means that the regulation of TPC and TFC by LEDs can be performed directly by inducing the expression of the key enzyme and indirectly by increasing the precursor molecule [95]. The results of the many different studies in the literature generally agreed on the role of BL and RL as stimulating LEDs, whilst GL was less effective [94,102,107] or ineffective [56]. Despite that Loi and colleagues did not detect a significant increase in phenolic compounds in broccoli [56], a positive effect was registered by Jin and colleagues, who used the same wavelength but a 2-fold intensity [108], proving that light intensity also plays an important role [95,109]. Generally, higher light intensities were more effective than low intensities in inducing light-responsive genes and increasing the polyphenol content in plants. Nonetheless, results also depend upon plant species [15,93], cultivars [101,110], and timing of LED exposure [111]. Most importantly, each polyphenolic compound has a specific response to light quality [91]. Thus, establishing whether light intensity is more determinant than light quality or vice versa is quite challenging; further efforts should be dedicated at designing experiments which encompass the use of different light qualities at different intensities and a comprehensive evaluation of the antioxidant compounds in specific cultivars. The most recent studies are reported in Table 3.

### 4.3. Photosynthetic Pigments

Chlorophylls (chls), carotenoids, and anthocyanins represent the main class of plant pigments in nature [114,115]. Chls are magnesium-tetrapyrrole pigments and are the most abundant and ubiquitous photosynthetic pigments distributed in higher plants, algae, and cyanobacteria. They have been classified into five groups (chl a, b, c, d, and f) based on the variations in five-membered ring structures or side chains [116]. All green plants contain chl a and b, both located within the thylakoid membrane of chloroplasts, whereas chl c, d, and the more recently identified chl f are present in algae and cyanobacteria. Chls biosynthesis and its regulation have been extensively investigated [117,118,119,120]. It is a complex pathway involving more than 17 enzymes and mainly subdivided into five parts: synthesis of 5-aminolevulinate acid (ALA), condensation of two ALA molecules to form a pyrrole ring (porphobilinogen, PBG), polymerization of four molecules of PBG to form a linear tetrapyrrole, conversion to the circular protoporphyrin IX (PPIX) via several decarboxylation and oxygenation reactions, and insertion of Mg to PPIX (more details in Figure S3).

Chls are green in color because they reflect the GL and absorb strongly in the red (625–675 nm) and blue regions (425–475 nm) of the visible spectrum. Chl a is the most abundant form which is present both in the reaction centers and in the light-harvesting complexes, whereas chlorophyll b is present in higher plants as a light-harvesting accessory pigment.

Carotenoids are lipophilic secondary metabolites, mainly belonging to the terpenoid group, and can be divided into two groups: carotenes, such as α-carotene, β-carotene, γ-carotene, and lycopene, and xanthophylls, such as β-cryptoxanthin, lutein, zeaxanthin, and astaxanthin. They are pigments abundantly produced in floral tissues and fruits, providing them with the orange, red, and yellow colors. Carotenoids are components of the light-harvesting complex in chloroplasts and can limit the damage to cell membranes caused by excess light. They are primarily involved in photosynthesis and photoprotection, as they absorb light in the blue, green, and violet regions. The majority of carotenoids are bound to the photosynthetic apparatus: they collaborate with chlorophylls in promoting light uptake and quenching the excess energy which would otherwise lead to the formation of the oxidant singlet oxygen (^1^O_2_) [17]. Xanthophylls can also provide photoprotection by the so-called non-photochemical quenching (NPQ) in which violaxanthin, antheraxanthin, and lutein epoxide are converted to zeaxanthin and lutein. This reaction promotes energy dissipation in the light-harvesting antenna proteins through harmless heat when excess energy is harvested [121]. In non-photosynthetic organelles, they act as antioxidants, color attractants, and precursors of plant hormones.

Indeed, plant hormones strigolactone and abscisic acid are produced from the xanthophyll violaxanthin, thus implying the indirect involvement of carotenoids in plant growth and development [122].

Light regulation of carotenoids is essential to counterbalance ROS production and to maintain the redox status of the cell. In fact, chloroplasts are one of the sites where ROS are produced the most [123]. Carotenoids are important players of the chloroplast redox homeostasis, acting both as antioxidants and as signal molecules in their oxidized forms [124].

The accumulation of carotenoids and other pigments is genetically driven in many plants and fruits. However, light plays a very important role in their biosynthesis and accumulation, having a stimulating effect [125]. 

Light quality and intensity, as well as temperature, and storage conditions highly influence the photosynthetic process through the content of photosynthetic pigments, [126,127,128]. As reported for the phenolic compounds, also the chlorophyll and carotenoid biosynthesis can be regulated by light-responsive genes [129,130,131].

With regard to chls, previous studies have identified differentially expressed genes in plants that are regulated by GL, BL, and RL [132,133,134]. In particular, Li and colleagues [134] showed that the content of chl into the leaves of grape plantlets grown under BL was significantly higher compared to the content in plantlets grown under WL, GL, or RL. The authors highlighted that the photosynthetic pigment contents and chloroplast development in the leaves of grape plantlets were notably promoted by BL but were inhibited by GL or RL. The results obtained from the RNA-seq analysis revealed that BL up-regulated eight key genes involved in the chl biosynthesis, including glutamate-1-semialdehyde 2, 1-aminomutase (HemL), hydroxymethylbilane synthase (HemC), uroporphyrinogen decarboxylase (HemE), coproporphyrinogen III oxidase (HemF), protoporphyrinogen oxidase (HemY), magnesium-protoporphyrin-O-methyltransferase, protochlorophyllide reductase, and chlorophyll(ide) b reductase (more details in Figure S3).

LEDs have been exploited to increase carotenoids and xanthophylls, such as β-cryptoxanthin, antheraxanthin, violaxanthin, and lutein. Despite having an important role in photoprotection, the increase in such compounds has also a commercial significance in many foods, such as citrus fruits, because of their importance in determining the external and internal coloration, which is an important commercial feature [135,136]. The enzyme phytoene synthase (PSY) is the rate-limiting enzyme in the carotenoid biosynthetic pathway; the PSY encoding gene is under the direct regulation of PIFs, which are degraded or activated by light [137]. Moreover, other carotenoid genes can be up-regulated by RL, such as lycopene-β-cyclase (LCYB), β-carotene hydroxylase (CHYB), and violaxanthin de-epoxidase (VDE) (Figure S4) [98]. BL has been frequently reported to be able to induce the expression of PSY and the accumulation of beta carotene in mandarin (*Citrus unshiu* Marc.) [135], baikal skullcap (*Scutellaria baicalensis*) [138], and different microgreens [109]. In contrast, some other studies showed that RL was more efficient than BL in increasing the carotenoids or chl contents [96,139,140,141], even though exposure to RL notably affects chl contents by down-regulating the tetrapyrrole precursor ALA [142]

Overall, most of the studies listed in Table 4 showed that the use of mixtures such as RL+BL [96,142,143,144,145,146] or fluorescent WL + RL or BL [147,148] has proven to be more efficient compared to monochromatic treatments. As regards GL and YL, very few studies report their use, with little or no significant results [56,139,142,149].

Limited data are available with regard to the effect of the irradiance levels on carotenoids accumulation. As reported for other antioxidants, the effects are not univocal and depend on the species, compound considered, and type of light [150]. Chl synthesis is notably affected by light intensity [151]. In a recent study, Loconsole and colleagues [152] highlighted that at the highest PPFD (194.54 µmol m^−2^·s^−1^), the content of chlorophyll and carotenoid was the lowest, while the opposite was observed for the lowest light intensity and shortest photoperiod. However, in a previous study, Samuoliené et al. [153] showed that increasing PPFD level led to an increase in the content of chlorophylls and carotenoids.

### 4.4. Glucosinolates

Glucosinolates (GLSs) are bioactive nitrogen- and sulfur-containing compounds occurring in some dicotyledonous angiosperms of the order Capparales, especially in the economically important Brassicaceae family [155,156]. Over 120 GLSs have been identified with a common β-D-thioglucosides N-hydroximinosulfates core structure and a variable side chain derived from amino acids [157]. They have been classified into three groups based on the structure of different amino acids precursors: aliphatic GLSs derived from alanine, leucine, isoleucine, methionine, or valine, aromatic GLSs derived from phenylalanine or tyrosine, and indole GLSs derived from tryptophan [156].

The pungent taste of the edible plants containing GLSs is mainly due to the presence of hydrolysis products of GLSs, including isothiocyanates, nitriles, oxazolidine-2-thiones, epithionitriles, and thiocyanates, formed when plants are stressed by biotic and abiotic factors. The degradation process begins with the hydrolysis of the thioglucoside linkage, catalyzed by the enzyme myrosinase, into glucose, sulfate, and an unstable aglycone, which then rearranges spontaneously into a wide range of active compounds depending on the structure of the side chain and the reaction conditions [149].

Several studies have shown that the GLSs hydrolysis products represent the bioactive component of these compounds. They are well-known for their involvement in plant defense response against insects and pathogens [158,159,160]. Moreover, several GLSs hydrolysis products may play a prominent role in chronic diseases prevention, including certain types of cancer, diabetes, cardiovascular disease, neurodegeneration, and several inflammatory disorders due to the oxidative stress [161,162,163,164]. The antioxidant properties of GLSs hydrolysis products were largely investigated. In particular, in vitro and in vivo studies have shown the ability of some GLSs hydrolysis products, generally associated with aliphatic isothiocyanate, to prevent cancer development through the induction of some detoxification enzymes (Phase II), including NAD(P)H quinone reductase, heme oxygenase 1, and glutathione S transferases [165].

GLSs, together with the phytochemicals mentioned above, have been reported to be affected by light of different wavelengths, and there is evidence that the effect of light to plant biomass production can influence the secondary metabolism in relation to GLS biosynthesis [2,166]. Various LEDs have been tested for their ability to influence GLSs contents, showing that their effects strongly depend on plant species and cultivar, plant organs, and GLS genotype [113,167,168,169].

Although the genetic bases of GSL biosynthesis were well investigated, little is known about the effect of different LED lights on the genes involved in GLS biosynthesis and its regulation. Some studies conducted on *Arabidopsis* and some other *Brassica* species revealed that GS-ELONG, GS-OX, GS-AOP, and GS-OH loci play an important role in GLS biosynthesis and content variability [170,171,172]. In 2001, Kliebenstein and colleagues [173] showed that the *AOP2* (GSL-ALK locus) and *AOP3* (GSL-OHP locus) genes, which map to the GSL-AOP locus, are responsible for converting methylsulfinyl alkyl glucosinolates into alkenyl and hydroxyalkyl glucosinolates, respectively. The presence or absence of either of the loci, as well as the functional or nonfunctional copy of those two genes, influences the type of glucosinolate produced in a defined species. Furthermore, in *Arabidopsis*, the expression of the *AOP2* gene was found to be up-regulated by light and highly expressed in the photosynthetic parts of the plant [174] (Figure S5). A recent study evaluated the effect of dark, white, red, and blue LEDs on the health-promoting phytochemicals and antioxidant capacity of Chinese kale sprouts [168]. The results showed that blue light, unlike the other light treatments, significantly decreased the content of gluconapin in shoots, while it increased the glucoraphanin content in roots. In aliphatic GLS biosynthesis, AOP2 catalyzes the conversion of glucoraphanin to gluconapin [172]. This might indicate that BL negatively affects the AOP2 activity, though further research is needed for a better understanding of the mechanism involved.

The most pronounced effect on the GLSs contents by LED lighting treatment is achieved by BL and RL, though few studies analyzed the impact of more LED wavebands on GLSs-containing plants [111,175].

When looking further at the individual experiments, effects varied across the analyzed species and tissues. For example, Kopsell and Sams [176] analyzed the concentrations of GLSs in broccoli microgreen tissues after exposure to RL/BL and BL treatments and found that the level of many aliphatic GLSs increased after exposure to the BL treatment, while indole GLSs were unaffected by the LED lighting treatment. Similarly, the level of aliphatic GSLs increased and that of indole GLSs was lower when plants of *Cardamine fauriei* were grown under BL irradiation [177]. However, in other studies, the BL treatment showed the opposite effect or no effect in GLSs biosynthesis [168,169,178,179].. Qian and colleagues [168] showed a significant reduction in aliphatic and indole GLSs contents in the shoots of Chinese kale sprouts, after exposure to BL treatment. Further, neither high blue ratio (31.7% BL/66.3% RL) nor low blue ratio (14.8% BL/81.3% RL) seemed to affect the GLSs content on different cultivars of rapeseed sprouts [169]. As suggested by some authors [2,176], light treatment might affect the side chain of the GLS rather than the core structure, and BL likely positively affects the amino acid involved in the aliphatic and aromatic GLSs biosynthesis, whereas it may have no effect on indole GLS (tryptophan-derived) biosynthesis. This hypothesis should be further investigated to reveal the exact mechanism of LED light on GLSs synthesis. The most recent studies on LED effects on GLS content in different plants are summarized in Table 5.

## 5. LED Lighting: Advantages and Challenges in Plant Growth and Postharvest Management

LED technology has grown tremendously on a global scale over the last decade, quickly replacing traditional lighting systems (incandescent, fluorescent, high-intensity discharge lamps, HID) in different fields, including the horticultural sector, as supported by a growing research community [21,180]. Furthermore, the strategic collaboration among LED manufacturers, plant producers, and academics is enabling extraordinary advances in the science and application of plant lighting, ranging from growth chambers to greenhouse application, vertical farming, and postharvest yield/crop management.

As mentioned above, higher energy efficiency, versatility, long lifetime, and cost saving features are some of the major advantages of LED lighting. Unlike HID and HPS, LEDs show reduced heat emission leading to prevent thermal degradation and optimizing space in indoor farming and storage management applications. Moreover, LED technology, compared to conventional lamps, enables a wide variety of spectral outputs and allows for controlling the directionality of light and regulating light intensity, all of which make it more suitable for the growth, preservation, and storage of fresh horticultural products [180,181,182].

**Figure 5:**
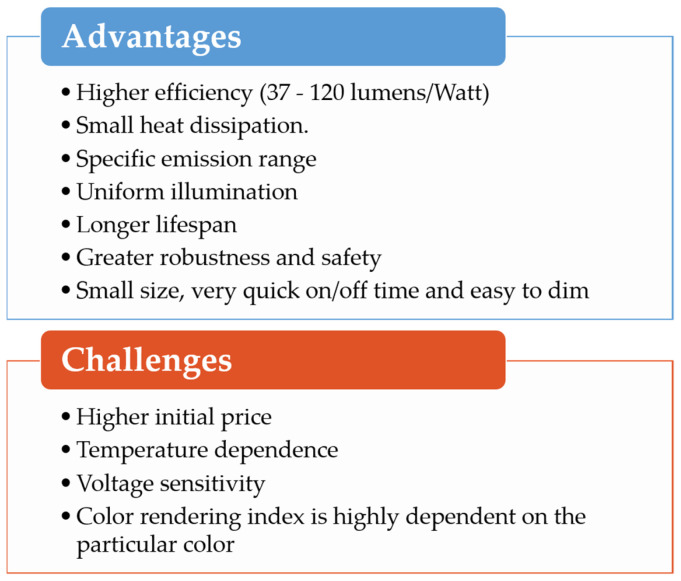
Advantages and challenges of LEDs.

In this review, we have focused on LEDs’ effects on some plant metabolites, with particular attention on the content of some antioxidant compounds both during the plant growth and at the postharvest level. Furthermore, specific light wavelengths and intensities may also affect the content of potentially harmful or undesirable molecules, reducing or improving the nutritional value of plants [55,183]. LED technology offers several advantages with respect to the conventional lighting systems; however, several challenges still have to be tackled, as reported in Figure 5.

Although several studies have addressed the benefits of LEDs for the growth of horticultural crops and the enhancement of their nutritional value, the findings about their effect on the quantity and quality of the plant crops in the postharvest stage are relatively recent and focused on few fruits and vegetables species [182,184]. There is evidence that LED lighting can influence the shelf life and quality of fresh produce, inhibiting weight loss, senescence, and over-ripening, and enhancing the production of antioxidant compounds [55,56,73,98,108,135]. Most of the results showed that BL promotes the accumulation of phytochemical constituents, including polyphenol in bananas (*Musa acuminate*) [100], Vit C in citrus juices (*Citrus unshiu* and *Citrus sinensis*) [72], Chinese cabbage (*Brassica campestris*) [75], and strawberry (*Fragaria ananassa*) [75], and anthocyanin in sweet cherries (*Prunus avium*) [84] and in Chinese bayberry fruit (*Myrica rubra*) [185]. In other studies, BL shows less effective results, compared to other light treatments. For instance, Song and colleagues [73] showed that RL was able to inhibit senescence of Pak-choi (*Brassica rapa* ssp. *Chinensis*) and to improve the content of chl, Vit C, and total soluble proteins, compared to BL and FR light. Similarly, Loi and colleagues [56] compared five light treatments during postharvest storage of broccoli (*Brassica oleracea* L. var. *italica*) (see Table 4) and revealed that chlorophyll content and TPC were higher under GL and RL treatments, respectively, while BL had no positive effects on Vit C levels.

Currently, RL and BL have been recognized as the most suitable treatments for plant growth and development of tailored food. However, there is a growing consensus that other LEDs, including YL or GL, may contribute, both monochromatically and combined, to promote higher biomass and yield or preserve plant quality [175,186,187]. In a recent review, Smith et al. [187] proved that supplemental GL to the LED light recipes may improve or maintain plant quality, yield, and water-use efficiency and contribute to photosynthetic carbon assimilation. They showed that unlike BL and RL, which are absorbed in the top and middle layers of leaf cells, respectively, GL is weakly absorbed by chlorophylls, and hence it can penetrate deeper and reach the bottom layer of cells owing to the phenomenon known as the detour effect [188]. However, the authors highlighted that not all green wavelengths have the same responses within the plant. For example, lettuce irradiated with different GL (peak wavelength at 510 nm, 520 nm, and 530 nm) and PPFD (100, 200, and 300 μmol m^−2^·s^−1^) showed distinct growth responses, even though the difference in peak wavelength among the GL was only 10 nm [189]. In particular, leaf elongation was stimulated more by GL at 530 nm, while GL at 510 nm and higher intensity promoted plant growth. This result, and some other evidence in the literature over the years, showed that, even with outstanding advancements in LED technology in the horticultural field, further improvements are needed, not only in lamp performance metrics, but also in experimental design.

Until recently, the insufficient availability of data on lamp performance metrics and quality standards did not help growers to compare results and LED options, driving confusion and lack of references. In 2017, the American Society of Agricultural and Biological Engineers (ASABE) published the S640 standards on the quantities and units used to describe horticulture lighting (ASABE, 2017) [190], including 33 new metric definitions for horticultural lighting, among which are photosynthetic active radiation (PAR), expressed as photosynthetic photon flux (PPF, PAR emitted by a source, measured in units of micromoles) and photosynthetic photon flux density (PPFD, PAR that falls on a unit of surface area). Although these new metrics are compatible with metrics previously defined, they are, however, specific to the needs of horticulture and plant biology. Furthermore, for the evidence that, outside the visible light (400–700 nm), plants respond to UV and FR radiation, the metrics are divided into three spectral ranges: photosynthetic (400–700nm), UV (100–400nm), and far-red (700–800). Moreover, the document allows describing radiation in terms of quantity of energy (measured in watts) or photons (measured in micromoles). Afterwards, in 2018, ASABE released the S642 standard, focusing on the performance of LEDs, arrays, and modules relative to the impact on plant growth and development (ASABE, 2018) [191]. Lighting manufacturers are adopting the metrics used in the horticulture sector. However, a recent review [192] showed that 30% of the LED lighting system producers included in the study still do not report the photosynthetic photon efficacy of lamps.

Besides the appropriate LED metrics implementation to the horticultural sector, there are some other challenges that need to be tackled, depending on the aim of the request (growth, postharvest, development). Among these, there are the influence of light intensity, irradiance, temperature, and power supply on the physiological and biochemical responses of the plant, and how those responses vary among species and within cultivars of the same species.

The monochromaticity property of LEDs limits the propagation of radiant heat, making them even more suitable for the horticultural sector, since they can avoid the harmful effects of radiant heat on the quality of agricultural commodities. Although LEDs are relatively efficient, about 65–70% of the supplied electric power generates heat instead of light because of low IQE and light extraction efficiency [193]. One of the major factors in determining the lumen output of an LED is the p-n junction temperature. As the temperature increases, the light intensity and LEDs lifetime decrease, and colorimetric properties are altered. The junction temperature is mainly influenced by three factors: environmental temperature, thermal resistance (defined as the rise in temperature of a component per unit of power dissipated) between the LED junction and its surrounding, and the driven current. For this reason, LED lamps generally require a proper cooling device and temperature-controlled environments [194] and this supports their use in refrigerators and in cold-chain storage or in transport vehicles.

A growing number of studies suggest that the overall quality of vegetables prior to or even after harvest is highly dependent not only on the spectral composition but also on LED intensity and photoperiod [195,196,197]. Jones-Baumgardt et al. [196] tested the effect of combined BL/RL (15:8 ratio) at six PPFD treatments on growth, yield, and quality of kale (*Brassica napus* L. ‘Red Russian’), cabbage (*Brassica oleracea* L.), arugula (*Eruca sativa* L.), and mustard (*Brassica juncea* L. ‘Ruby Streaks’), demonstrating that arugula and mustard exhibited greater levels of phenotypic plasticity to light intensity (600 µmol m^−2^·s^−1^) than kale and cabbage. Furthermore, low intensity seemed to improve the postharvest quality of fruits and prevent senescence during storage [198]. Light distribution and irradiance uniformity are also important parameters to be evaluated because the photosynthetic properties depend on the leaf age and/or distance between the lighting device and plant canopy; therefore, a different response might be revealed in the lower canopy compared to the upper leaves [181,199]. Moreover, since the intensity of light radiation that reaches a surface is inversely proportional to the square of the surface’s distance from the source, light levels vary and are inconsistent as plants grow, which suggests that light output could be modified according to the plant photosynthetic requirements.

Overall, understanding the physiological responses induced by LED lights is a crucial step to regulate plant morphogenesis, enhance the nutritive value of crops, and preserve quality in postharvest fresh products. However, data from the literature are often contradictory because, over the years, many research projects focused their efforts on a few selected species or cultivars, and little is known about the comparison among closed species, more cultivars, or types of the same cultivar [22,169,200,201]. Eventually, further efforts should be devoted to including harmonized LED metrics and to comparing different light qualities and intensities in the same experimental design.

## 6. LEDs as a Tool to Improve Microbiological Food Safety

Food safety in the food chain is the basis for an effective functioning of national and international markets with trust and transparency. Food safety risks occur along the food chain due to different biological and chemical contaminants, and the development of novel methods for decontamination of food and food processing remains a major global problem that causes significant social, as well as economic, efforts.

The thermal techniques are the most efficient and used methods to eliminate pathogens, but their application cannot be applied to certain types of foods, such as fresh produce in ready-to-eat salads and so on.

Light is crucial for many organisms and can be influential for many cellular processes, being important for survival, growth, and reproduction [202].

Light induces cell damage, injury, and death in microorganisms through several mechanisms, which are related to the presence of photosensitive endogenous compounds such as porphyrins and flavins. The absorption of the light at specific wavelengths by these molecules promotes them to excited energy states [203] that can induce photodynamic reactions and locally generate ROS. The formed ROS then react with multiple cellular components, further causing microbial cell death by oxidative damage [204]. Experiments on various bacteria show that BL or near-UV radiation has been found to be most effective in inactivating cells, while RL has minimal efficacy. In particular, LEDs within the band of 400 to 450 nm, with a peak wavelength of 405 nm, are most effective, as the peak coincides with the absorption maximum of porphyrins [205,206,207]. The susceptibility of bacteria to the light appears to vary widely among species and depending on the exposure time.

Molecules with either antioxidant or ROS-generating functions are particularly interesting to investigate as these could provide an indication to explain the differences in inactivation rates among the different or the same species of bacteria. Kumar et al. [208] hypothesized that a host of other factors such as other inactivation pathways and microbial responses to light treatment may play critical roles in determining the extent of inactivation for a particular species.

In fungi, light is one of the most important signals, since it influences several physiological responses such as secondary metabolism, pigmentation, sexual development, asexual conidiation, and the circadian clock. The sensing of light in fungi is over a broad spectrum range, from UV to FR light. A variety of photoreceptors are conserved in fungi and the range of perceptible light intensities covers more than ten orders of magnitude [202]. The first knowledge about the influence of light on the growth and physiology of fungi was obtained on *Neurospora crassa* that has become the most prominent model system for studying mechanisms of photoreception, circadian rhythms. Moreover, in *N. crassa* light perception and the response to BL incubation [209,210] have been elucidated at the molecular level. The white-collar complex is the first and best studied photoreceptor system in fungi. However, the increasing number of fungal genome sequences revealed the presence of a number of different photoreceptors in each fungus and suggests fascinating but rather complex and diverse regulatory systems.

The effect of LED light has been studied to prevent fungal infection of fruits in postharvest storage. In general, BL at a moderate intensity was able, depending on fungal species, to dramatically reduce the soft rot area, mycelial growth, and sporulation of various fungi (*Penicillium digitatum*, *P. italicum*, and *Phomopsis citri*), on the surface of fruits compared with WL and darkness [211,212,213,214]. Additionally, in citrus fruit, BL was able to induce changes in ethylene production and phenolic compounds that are important players in the defense of citrus fruit against *P. digitatum* [215,216,217,218,219]. However, the molecular mechanism of *P. digitatum* resistance remains unknown [220].

Yu and Lee [221] studied an interesting application of RL (645 nm) that may be useful in overcoming limitations that LED irradiation possesses. The application of RL (645 nm) on the surface of the fruit was more effective in enhancing the antifungal effect of *Bacillus amyloliquefaciens* by enhancing the motility and biofilm formation at 240 μmol m^−2^ s^−1^ compared with other wavelengths. The cell-free supernatant of bacteria irradiated with RL revealed a higher production of iturin and fengycin, which are antifungal lipopeptides. This synergistic application of antagonistic bacteria and LED irradiation by stimulating the growth of antagonistic biofilms might prevent fungal growth on fruits after LED irradiation is stopped. In fact, it is reported that LEDs do not reach the inner tissues of the plant; thus, they are not able to completely inhibit fungal growth [211].

Fungi are potent producers of secondary metabolites, such as antibiotics or mycotoxins [222,223], and light also controls the production of those metabolites. In particular, mycotoxins produced by toxigenic fungi represent a great concern worldwide either for the economic implications or for the health of the consumers. Light modulates mycotoxin production with a promoting or an inhibiting effect depending on the species and could be a new physical parameter to control and degrade the production of these metabolites, at least for surface-growing fungi. Some toxigenic fungi such as the *Fusaria* or *Alternaria* genera seem to be resistant to various light wavelengths. However, the production of secondary metabolites (e.g., mycotoxins) was reduced after the application of BL [224,225]. In fact, BL was the most effective in reducing ochratoxin biosynthesis, with results depending on the species treated. A clear correlation exists between the intensity of BL and the inhibitory effect. Weak royal BL led to a moderate reduction in ochratoxin A for *Penicillium verrucosum*, *P. nordicum*, *Aspergillus steynii*, and *A. carbonarius*. Higher intensities of royal BL were shown to reduce ochratoxin A biosynthesis to either non-detectable (*P. verrucosum, A. steynii*) or to very low levels (*A. carbonarius, P. nordicum*) [224].

Light could represent a new resource against mycotoxin contamination, which can be developed in a targeted way, with limited costs and easily integrated in food processing and preservation.

## 7. Perspectives 

**Figure 6:**
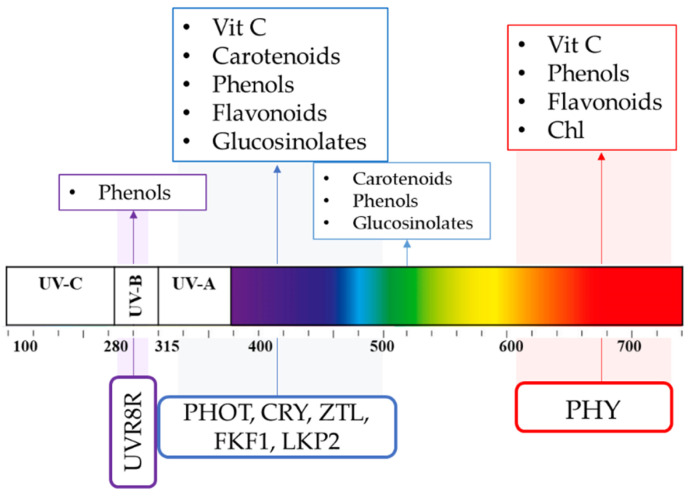
Photoreceptor-mediated effects on plant antioxidant compounds, namely, vitamin C, carotenoids, phenols, chlorophylls, flavonoids, and glucosinolates. Higher font dimension indicates a higher modulation. Ultraviolet radiation A (UV-A), ultraviolet radiation B (UV-B), ultraviolet radiation C (UV-C), phytochrome (PHY), cryptochrome (CRY), phototropin (PHOT), flavin binding Kelch domain F box protein (FKF1), zeitlupe (ZTL), LOV Kelch protein2 (LKP2), and UV-B resistance locus 8 (UVR8).

LED technology provides unique advantages over the conventional lighting systems and proves to be extremely versatile. LEDs have the potential to regulate several aspects of plant growth, development, the accumulation of different secondary metabolites (Figure 6) [182], and the overall plant metabolome [226].

The positive effect on the antioxidant compounds of vegetables has several implications, which go beyond the simple nutraceutical value of the food. An increased antioxidant capability endows the plants with a higher resistance towards harsh environmental conditions (excessive light, drought, extreme temperatures), toxic pollutants, and pathogens [107,227]. Indeed, many secondary metabolites have been shown to possess antimicrobial and antifungal properties [228]. Light-induced resistance may also arise from the induction of defense genes and the salicylic acid pathway-mediated systemic acquired resistance [229]. This may potentially lead to a reduced need for chemical treatments in the field [230]. In addition, the use of LEDs in the postharvest can prolong the shelf life, delay senescence [55,111], and, thus, reduce food waste.

Senescence is an unwanted phenomenon which leads to the loss of the nutritional and commercial value of the product. Generally, weight loss, oxidation, loss of pigments, yellowing in green vegetables due to chl degradation, and alteration of transpiration and respiration processes characterize senescence [182]. BL, RL, and GL can directly affect pigment accumulation, the antioxidant status, and photosynthesis, thus slowing senescence and extending the shelf life of fruits and vegetables.

LEDs can be a powerful tool to enhance plant productivity of protected cultures, which are grown with alternative methods to conventional agriculture, such as soilless, aeroponics, or hydroponic systems. For example, strawberry (*Fragaria* L.) is one of the fruits which is cultivated in controlled environments because of the high economic income they provide, especially early during the season. BL and RL were effectively employed to increase plant biomass and fruit quality [231,232]. Nonetheless, the implementation of LED systems in artificial cultivation environments can provide unique advantages in terms of sustainability, less land usage, and in the future scenario of climate change [233]. 

Another important implication that has been explored only recently is the impact of LED treatment on the sensory profile of off-season products. The increase in sugars, acids, minerals, and other components due to LED treatment can significantly impact the sensory profile of fruits and vegetables and improve the consumers’ acceptance [234].

**Figure 7:**
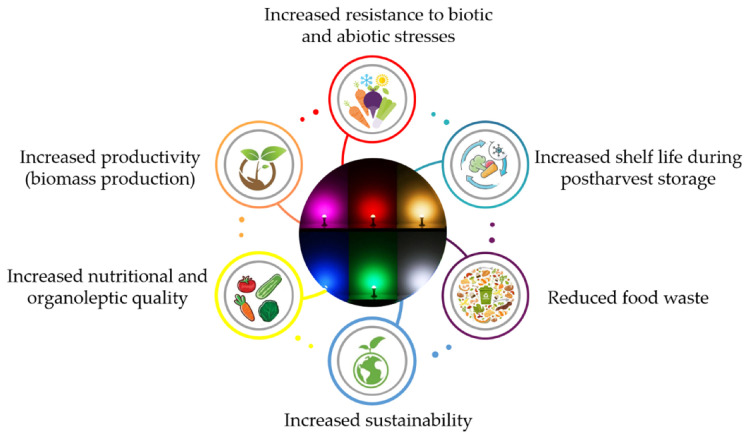
Future perspectives and positive outcomes of LEDs for growth and postharvest storage of food commodities. Further details are given in the text.

Even though LEDs found wide application in small-scale and greenhouse production, only few applications in house refrigerators are present currently in the market. In most of the cases, BL, GL, and WL are used to increase vitamin C content, sustain the photosynthetic process, and preserve chlorophyll from oxidation. LEDs application in food science is still in its early days and many possible positive outcomes can be envisioned in the near future (Figure 7).

## 8. Conclusions

In conclusion, LED technology has shown great potential to promote the growth and the synthesis of beneficial compounds and prolong the shelf life of fruits and vegetables during postharvest storage. So far, a comparison of the studies in the literature is challenging because of the different experimental designs, plant species and cultivars, light types and intensities, and other environmental parameters which are not always fully disclosed or harmonized. Additionally, only recently have quality standards been introduced.

A deeper knowledge of the spectral-dependent responses at the molecular level and the role of photoreceptors can be performed in controlled environments by means of an integrated approach, based on transcriptomics, proteomics, and metabolomics. Data on the plant–LED interaction effects are already available in the literature and, due to the interest of the scientific community, in the next years, a huge amount of data will be continuously added. These data can be analyzed to extrapolate and correlate different types of LED treatment with the fitness of the plant and its antioxidative profile. In this context, artificial intelligence and machine learning algorithms will allow us to predict the plant health and the shelf life of postharvest horticultural crops. Further improvements and studies are therefore essential to design specific LED protocols and enable us to exploit this technology at its fullest potential.

## Figures and Tables

**Table 1:** The most representative characteristics for some commercially used LED semiconductors.

Materials	Formula	Wavelengths [nm]	Light Color	Forward Voltage [V]
Gallium-Phosphide Aluminium-Gallium-Arsenide Gallium-Arsenide-Phosphide Aluminium-Gallium-Indium-Phosphide	GaP AlGaAs GaAsP AlGaInP	610 < λ < 770	Red	1.6–2.0
Gallium-Phosphide Gallium-Arsenide-Phosphide Aluminium-Gallium-Indium-Phosphide	GaP GaAsP AlGaInP	590 < λ < 610	Orange	2.0–2.1
Gallium-Arsenide-Phosphide Gallium-Phosphide	GaAsP GaP	570 < λ < 590	Yellow	2.1–2.2
Gallium-Phosphide Aluminium-Gallium-Phosphide Aluminium-Gallium-Indium-Phosphide	GaP AlGaP AlGaInP	500 < λ < 570	Green	1.9–4
Silicon carbide as substrate Zinc Sulfide	SiC ZnS	450 < λ < 500	Blue	2.4–3.7
Gallium-Nitride Indium-Gallium-Nitride	GaN InGaN	400 < λ < 450	Violet	2.7–4.0
Blue diode with yellow phosphor		Broad spectrum	White	3.5

**Table 2:** LED effects on the vitamin C (Vit C) content of different plants. Blue light (BL), red light (RL), green light (GL), yellow light (YL), white light (WL).

Plant Species	LED (Wavelength, Intensity), Photoperiod, and Experimental Parameters	Type of Application	Effect	Reference
Satsuma mandarin (*Citrus unshiu* Marc.), Valencia orange (*C. sinensis* Osbeck), Lisbon lemon (*C. limon* Burm.f.)	BL (470 nm, 50 μmol m^−2^·s^−1^) + RL (660 nm, 50 μmol m^−2^·s^−1^) for 4 weeks; BL (50 and 100 μmol m^−2^·s^−1^) for 4 weeks; continuous and pulsed BL (100 μmol m^−2^·s^−1^) for 4 weeks	Postharvest	BL increased Vit C content RL did not affect Vit C content	[72]
*Brassica rapa* ssp. *Chinensis*	RL (35 μmol m^−2^·s^−1^) for 8 h/per day at 20 °C	Postharvest	RL increased Vit C, RL delayed senescence, BL no effect	[73]
*Brassica oleracea* L. Var. *Italica*	BL (470 nm, 50 μmol m^−2^·s^−1^) at 20 °C and RH > 95%, Modified WL (RL 660 nm > BL)	Postharvest	RL delayed senescence, modified WL (RL>BL) increased Vit C, BL no effect	[55]
*Fragaria ananassa* Duch. Cv. Fengguang	BL (470 nm, 40 μmol m^−2^·s^−1^) for 12 days at 5 °C, RH 80–90%.	Postharvest	BL increased Vit C	[75]
*Lactuca sativa* L. Cv. Lvdie	WL (80 RL + 20 BL, 250 μmol m^–2^·s^–1^)	Growth	5 GL + 80 RL + 15 BL decreased Vit C	[76]
Lettuce plants	25 RL + 75 BL (200 μmol m^–2^·s^–1^) 12 days under continuous irradiation); 23 °C (RH 50–60%)	Growth	25 RL + 75 BL increased Vit C	[77]
*Brassica campestris* L	BL + RL (B/R = 1:8) (80 μmol m^–2^s^–1^) 12-h photoperiod; 24–26°C; RH 40–50%	Seedlings	BL + RL increased Vit C	[78]
Lentil (*Lens esculenta* Moenh), wheat (*Triticum aestivum* L.), radish (*Raphanus sativus* L.)	GL (510 nm,100 μmol m^−2^·s^−1^; 12-h photoperiod; 27 °C) for lentil and wheat; GL + Amber Light for radish	Postharvest	GL increased Vit C (lentil and wheat); GL + Amber Light increased Vit C (radish)	[81]
*Solanum lycopersicum*	WL (263 μmol m^−2^·s^−1^) for 13 days nm	Postharvest	WL increased Vit C (plus 32%)	[82]
*Lactuca sativa* L. Var. *Capitata*	BL (480 nm), GL (520 nm), RL (630 nm), WL (455 and 570 nm), 400 μmol m^−2^·s^−1^	Postharvest	supplemented GL either 4 days or 43 days enhanced Vit C inside the crisp head lettuce	[83]
Apple (*Malus domestica* ‘Granny Smith’), red tomato (*Solanum lycopersicum* L.), bell pepper (*Capsicum annuum*)	Constant YL (590 nm, 1.81 W/m^2^) 10 °C for 7 days	Postharvest	YL not significantly increased Vit C	[84]
*Brassica rapa* ‘Dongdori’	WL, GL (524 nm), BL (436 nm), RL (664 nm) stored at 4–5 °C for 18 days	Postharvest	BL increased Vit C with BL > WL > GL > RL > non-irradiate	[85]

**Table 3:** LED effects on the polyphenol content of different plants. Blue light (BL), red light (RL), green light (GL), yellow light (YL), white light (WL), blue-violet light (BVL), fluorescent light (FL), white fluorescent light (WFL), high-pressure sodium light (HPS), ultraviolet B radiation (UV-B), ultraviolet A radiation (UV-A), white, blue, and green light combination (WBG), total phenolic compounds (TPC), total flavonoid compounds (TFC).

Plant Species	LED (Wavelength, Intensity), Photoperiod, and Experimental Parameters	Type of Application	Effect	Reference
Broccoli (*Brassica oleracea* L. Var. *Italica*)	BL (467 nm, 21 µmol m^−2^·s^−1^), GL (522 nm,24 µmol m^−2^·s^−1^), YL (587 nm, 27 µmol m^−2^·s^−1^) RL (625 nm, 66 µmol m^−2^·s^−1^) WL (3000 K color temperature, 40 µmol m^−2^·s^−1^); continuous irradiation at 4 °C	Postharvest	RL > YL > BL increased the TPC throughout the storage period	[56]
Brussels sprouts (*Brassica oleracea* var. *gemmifera*)	UV-B (300 nm, 0.02 µmol m^−2^·s^−1^), UV-A (365 nm, 14 µmol m^−2^·s^−1^), BL (470 nm, 11 µmol m^−2^·s^−1^), RL (660 nm, 2 µmol m^−2^·s^−1^); 16-h photoperiod	Sprouting	Compound specific effects	[91]
Buckwheat sprouts (*Fagopyrum esculentum Moench*)	BL (460nm), RL (625nm), FL (35 µmol m^−2^·s^−1^); 16 h of light; supplement and 8 h of dark per day	Sprouting	BL > RL > FL increasedTPC, TFC, and antioxidant capacity using ABTS radical cation assay. BL increased orientin, isoorientin, vitexin, isovitexin, rutin, and quercetin-3-O-robinobioside	[92]
Basil (*Ocimum basilicum*), arugula (*Eruca sativa*), and bloody dock (*Rumex sanguineus*)	BL (450 nm, 300 µmol m^−2^·s^−1^), BVL (440–420 nm, 300 µmol m^−2^·s^−1^), HPS (600nm, 400 W);	Growth	BL and BVL increased the phenolic acid content in basil, BV the flavonoids in arugula. No increase was registered in bloody dock with either BL or BV	[93]
Cherry tomato seedlings (*Solanum lycopersicum* L. ‘Cuty’)	RL (655 nm, 200 µmol m^−2^·s^−1^), BL (456 nm, 200 µmol m^−2^·s^−1^), GL (518 nm, 200 µmol m^−2^·s^−1^), WL (456 nm, 200 µmol m^−2^·s^−1^)	Growth	BL > WL > RL > GL increased the TPC and TFC	[94]
Tea (*Camellia sinensis* cv. Fujian Shuixian)	BL (450nm) at low (50 µmol m^−2^·s^−1^) medium (100 µmol m^−2^·s^−1^) and high (200 µmol m^−2^·s^−1^) intensity, WL (100 µmol m^−2^·s^−1^); 12-h photoperiod, 14 days	Growth	High-intensity BL down-regulated several genes involved in flavonoid biosynthesis; no effects elicited by low- and medium-intensity BL. BL increased the levels of 3′,5,6-trihydroxy-3,4′,7,8-tetramethoxyflavone 3-glucoside, galangin 3-[galactosyl-(1-4)-rhamnoside], and neocarthamin, and reduced the levels of 3-(2-caffeoylsophoroside) 7-glucoside, quercetin 3-(4‘-acetylrhamnoside) 7-rhamnoside, and spinacetin 3-(2‘- feruloylgentiobioside	[95]
Navel oranges (*Citrus sinensis Osbeck*)	UVC (10–280 nm, 100 µmol m^−2^·s^−1^), UVB (270–315 nm, 100 µmol m^−2^·s^−1^), UVA (315–400 nm, 100 µmol m^−2^·s^−1^), BL (470 nm, 200 µmol m^−2^·s^−1^), RL (660 nm, 150 µmol m^−2^·s^−1^), WL (100 µmol m^−2^·s^−1^); Continuous irradiation for 6 days; sampling after 0, 6, and 15 days	Postharvest	BL and RL retained more PC during the irradiation period, while WL, UVB, and UVC stimulated their accumulation after the irradiation period. RL and BL maintained the levels of diosmin, diosmetin 6,8-di-C-glucoside, hesperidin, didymin, neoeriocitrin, and narirutin; all Uvs increased narirutin, neoeriocitrin, and didymin	[98]
Sweet pepper (*Capsicum annuum* L cv. California Wonder, King of the North, Citrine F1 Hybrid)	RL (660 nm, 150 µmol m^−2^·s^−1^) BL (450 nm, 100 µmol m^−2^·s^−1^); 8-h photoperiod	Postharvest	RLwas more effective than BL in increasing the TFC, and PAL activity	[101]
Okra (*Abelmoschus esculentus* L.)	RL (630 nm, 17.28 W/m^2^), BL (470 nm, 17.28 W/m^2^), GL (560 nm, 17.28 W/m^2^); 8-h photoperiod	Postharvest	WL and BL increased the TFC content and the activity of key enzymes involved in the biosynthesis of phenolics	[102]
Bananas (*Musa acuminata* cv. Berangan)	BL (464–474 nm, 3920 µmol m^−2^·s^−1^), GL (515–525 nm, 4340 µmol m^−2^·s^−1^) equivalent to photosynthetic intensity of 100 W m^−2^, RL (617–627 nm, 5200 µmol m^−2^·s^−1^); continuous irradiation for 8 days	Postharvest	BL was more effective than RL and GL in increasing in TPC	[107]
Broccoli (*Brassica oleracea* L. Var. *Italica* cv. Chaoda No. 1)	WFL (300–700nm, (12–13 µmol m^−2^·s^−1^), GL (520 nm, 12–13 µmol m^−2^·s^−1^); 12-h photoperiod	Postharvest	GL increased the TPC	[108]
Red leaf lettuce (*Lactuca sativa* L., cv. Sunmang) and green leaf lettuce (*Lactuca sativa* L., cv. Grand Rapid TBR)	Various combinations of BL (456 nm) and RL (655 nm) BL: RL = 0:100%, 13%:87%, 26%:74%, 35%:65%, 47%:53%, 59%:41%, (171 µmol m^−2^·s^−1^), 12-h photoperiod	Postharvest	Intermediate BL/RL ratios (35%:65% > 47%:53% > 59%:41% in cv. Sunmang) stimulated the accumulation of TPC	[110]
Pea sprouts (*Pisum sativum* L.)	RL (635 nm), BL (460 nm), YL (585 nm), WL (30 µmol m^−2^·s^−1^); 12-h photoperiod	Sprouting	BL was more effective than WL, RL, and YL in increasingd the TPC and TFC with respect to the dark. BL increased the content of chlorogenic acid, *p*-hydroxybenzoic acid, caffeic acid, gallic acid, *p*-coumaric acid, ferulic acid, rutin, and resveratrol; WL increased the content of chlorogenic acid, *p*-hydroxybenzoic acid, caffeic acid, gallic acid, ferulic acid, rutin, and resveratrol; RL increased the content of *p*-hydroxybenzoic acid, caffeic acid, *p*-coumaric acid, ferulic acid, and rutin; YL increased *p*-hydroxybenzoic acid, caffeic acid, gallic acid, *p*-coumaric acid, ferulic acid, rutin, and resveratrol.	[112]
Canola sprouts (*Brassica napus* L.)	WL (380 nm, 50 µmol m^−2^·s^−1^), BL (470 nm, 50 µmol m^−2^·s^−1^), RL (660 nm, 50 µmol m^−2^·s^−1^), BL + RL; 16-h photoperiod	Sprouting	BL was more effective than WL, BL + RL, and RL in increasing the content of phenolic acids, flavonoids, and catechin. BL increased the content of benzoic acid, (+)-catechin, caffeic acid, and (−)-epicatechin; WL increased the contents of rutin.	[113]
Lettuce (*Lactuca sativa* L. Cv. ‘Grizzly’	WL (380–760 nm, 300 µmol m^−2^·s^−1^), RL (650–665 nm, 300 µmol m^−2^·s^−1^), BL (460–475 nm, 300 µmol m^−2^·s^−1^) or RL + BL (70%:30%), 14-h photoperiod	Growth	WL > BL increased the TPC	[96]
Longan (*Dimocarpus longan* Lour.)	BL (457 nm, 32 µmol m^−2^·s^−1^), GL (515 nm, 32 µmol m^−2^·s^−1^), WL 32 µmol m^−2^·s^−1^), and RL (660 nm, 32 µmol m^−2^·s^−1^) 12-h photoperiod; different BL intensities (16, 32,64, 128, and 256 µmol m^−2^·s^−1^), 12-h photoperiod; BL at 32 µmol m^-2^·s^-1^ with different photoperiods: 8, 12, 16, 20, and 24 h	Growth	BL was more effective than GL and WL in increasing the TFC; 32 µmol m^−2^·s^−1^ was the most effective intensity to increase the TFC; 12-h photoperiod was the most effective in increasing TFC; BL promoted the accumulation of epicatechin, but inhibitedthe synthesis of rutin.	[99]
Sarcandra Herb *(Sarcandra glabr*a)	WL (380–760 nm, 80 µmol m^−2^·s^−1^), RL, 656 nm, 80 µmol m^−2^·s^−1^), BL, 450 nm80 µmol m^−2^·s^−1^); 16-h photoperiod	Growth	BL increased the levels of cinnamic acid, 4-coumaric acid, chalcone, naringenin quercitin,kaempferol, and rutin, while it reduced the caffeic acid content. BL increased the expression of of key enzymes involved in the biosynthesis of phenolics (PAL, FLS)	[97]
Van’sweet cherry (*Prunus avium* L.)	UV-B (310nm, 0.046 W m^−2^), BL (444 nm, 1 W m^−2^), WBG composed by BL (470nm), GL (520 nm) and WL with a total 3.6 radiant flux W m^2^	Postharvest	BL increased the content of cyanidin 3-O-glucoside, cyanidin 3-O-rutinoside; BL was more effective than WBG in increasing PAL activity	[100]

**Table 4:** LED effects on the photosynthetic pigments of plants, chlorophyll (chl), and carotenoids. Blue light (BL), red light (RL), green light (GL), yellow light (YL), white light (WL), far-red light (FRL), fluorescent light (FL), white fluorescent light (WFL), blue fluorescent light (BFL), red fluorescent light (RFL), ultraviolet A radiation (UV-A), ultraviolet B radiation (UV-B), ultraviolet C radiation (UV-C).

Plant Species	LED (Wavelength, Intensity), Photoperiod, and Experimental Parameters	Type of Application	Effect	Reference
Chinese cabbage seeds (*Brassica campestris* L. Teaiqing)	YL (590 nm, 150 µmol m^−2^·s^−1^), GL (520 nm, 150 µmol m^−2^·s^−1^), RL (658 nm, 150 µmol m^−2^·s^−1^), BL (460 nm, 150 µmol m^−2^·s^−1^), RL + BL (6:1 ratio); 12-h photoperiod	Growth	All LEDs except RL + BL led to a decrease in total chl and carotenoids	[142]
Lettuce (*Lactuca sativa* L. cv. ‘Grizzly’	WL (380–760 nm, 300 µmol m^−2^·s^−1^), RL (650–665 nm, 300 µmol m^−2^·s^−1^), BL (460–475 nm, 300 µmol m^−2^·s^−1^) orRL + BL (70%:30%), 14-h photoperiod	Growth	RL + BL > RL > BL increased the total chl; RL+BL increased the total carotenoids. All LEDs improved the photosynthetic rate	[96]
Grape (*Vitis vinifera* L. × *V. labrusca* L cv. Summer Black)	RL (660nm, 100 µmol m^−2^·s^−1^), BL (460nm, 100 µmol m^−2^·s^−1^), RL + BL (660 + 630 + 460 nm)	Growth	BL increased the photosynthetic rate	[130]
Satsuma mandarin (*Citrus unshiu* Marc.)	BL (470 nm, 50 µmol m^−2^·s^−1^), RL (660 nm) 50 µmol m^−2^·s^−1^); continuous irradiation for 6 days at 20°C	Postharvest	BL increased the expression of carotenoid biosynthetic genes and carotenoids content	[135]
Pak choi (*Brassica rapa* ssp. *chinensis* cv. Black Behi)	BL (453nm, 80 µmol m^−2^·s^−1^), RL (633nm, 80 µmol m^−2^·s^−1^), WL (404–789nm, 80 µmol m^−2^·s^−1^); 12-h photoperiod	Growth	WL > RL > BL increased the carotenoid and chlorophyll contents	[140]
Sweet oranges (*Citrus sinensis* (L.) Osbeck)	RL (660 nm, 150 µmol m^−2^·s^−1^), BL (470 nm, 200 µmol m^−2^·s^−1^), WL (100 µmol m^−2^·s^−1^), UVA (315–400 nm, 100 µmol m^−2^·s^−1^), UVB (270–315 nm, 100 µmol m^−2^·s^−1^), UVC (100–280 nm, 100 µmol m^−2^·s^−1^); continuous irradiation for 6 days	Postharvest	Dark > RL increased the carotenoid content	[154]
Mustard (*Brassica juncea* L., ‘Red Lion’), beet (*Beta vulgaris* L., ‘Bulls Blood’), and parsley (*Petroselinum crispum Mill.*, ‘Plain Leaved or French)	Different combinations of BL (447 nm, 300 µmol m^−2^·s^−1^) RL (638nm, 300 µmol m^−2^·s^−1^), FRL (731 nm, 300 µmol m^−2^·s^−1^); 16-h photoperiod	Growth	BL led to the accumulation of chlorophylls, carotenoids, carotenes, lutein, violaxanthin, and zeathin	[109]
Broccoli (*Brassica oleracea* L. var. *italica*)	BL (467 nm, 21 µmol m^−2^·s^−1^), GL (522 nm, 24 µmol m^−2^·s^−1^), YL (587 nm, 27 µmol m^−2^·s^−1^), RL (625 nm, 66 µmol m^−2^·s^−1^), WL (3000 K color temperature, 40 µmol m^2^·s^−1^); continuous irradiation at 4 °C	Postharvest	GL increased the chlorophyll content; no effect on carotenoids elicited by all LEDs	[56]
Green Oak Leaf lettuce (*Lactuca sativa* var. *crispa*)	WFL (400–700 nm, 133 µmol m^−2^·s^−1^), BL (460 nm, 133 µmol m^−2^·s^−1^), RL (630 nm, 133 µmol m^−2^·s^−1^), FBL (WFL + BL), FRL (WFL + RL); 14-h photoperiod	Growth	FLR > FLB > RB increased total chlorophyll content and total carotenoids	[147]
Green Oak Leaf lettuce (*Lactuca sativa* var. *crispa*)	WFL + FRL (850 nm, 135 µmol m^−2^·s^−1^), WFL + RL (660 nm, 135 µmol m^−2^·s^−1^), WFL + YL (596 nm, 135 µmol m^−2^·s^−1^), WFL + GL (522 nm, 135 µmol m^−2^·s^−1^), WFL + BL (450 nm, 135 µmol m^−2^·s^−1^)	Growth	WFL + RL and WFL + BL increased the total chlorophyll and carotenoid content	[148]
Baikal skullcap (*Scutellaria baicalensis*)	RL (660 nm, 50 µmol m^−2^·s^−1^), BL (470 nm, 50 µmol m^−2^·s^−1^), WL (380 nm, 50 µmol m^−2^·s^−1^); 16-h photoperiod	Growth	BL > RL > WL increased the expression of carotenoids photosynthetic genes; BL increased the zeaxanthin, β-carotene, and 9-cis-β-carotene content	[138]
Green Chili (*Capsicum annuum* L.)	RL (660 nm, 50 µmol m^−2^·s^−1^), BL (470 nm, 50 µmol m^−2^·s^−1^), continuous irradiation for 3 days	Postharvest	RL induced the expression of lycopene-β-cyclase (Lcyb), β-carotene hydroxylase (CrtZ), and capsanthin/capsolubin synthase (Ccs); BL induced the expression of PS; RL > BL increased the carotene, free-capsanthin, and total carotenoid contents	[141]

**Table 5:** LED effects on the glucosinolate (GLS) content of different plants. Blue light (BL), red light (RL), green light (GL), white light (WL), far-red light (FRL), fluorescent light (FL), white fluorescent light (WFL), high blue ratio (HB), low blue ratio (LB).

Plant Species	LED (Wavelength, Intensity), Photoperiod, and Experimental Parameters	Type of Application	Effect	Reference
Broccoli (*Brassica oleracea* L. var. *italica* cv. Chaoda No. 1)	WFL (300–700 nm, (12–13 (µmol m^−2^·s^−1^)GL (520 nm, 12–13 µmol m^−2^·s^−1^) 12-h photoperiod	Postharvest	GL increased GLSs contents	[108]
Sprouting Canola (*Brassica napus* L.)	WL (380 nm, 50 µmol m^−2^·s^−1^) BL (470 nm, 50 µmol m^−2^·s^−1^) RL (660 nm, 50 µmol m^−2^·s^−1^) BL + RL; 16-h photoperiod	Growth	BL + RL determined the lowest levels of total GLSs. RL increased the level of sinigrin, glucobrassicin, and 4-methoxy glucobrassicin, while BL increased the level of glucoraphanin. WL and BL increased the level of glucoalyssin and gluconapin	[113]
kale sprouts (*Brassica oleacea* var. *alboglabra* Bailey)	WL (440-660 nm, 30 µmol m^−2^·s^−1^) RL (660 nm, 30 µmol m^−2^·s^−1^) BL (470 nm, 30 µmol m^−2^·s^−1^). 16-h photoperiod	Growth	No effects elicited by WL and RL; BL inhibited the accumulation of GLSs in shoots.	[168]
white- and yellow-flowering rapeseed (*Brassica napus* L.)	HB (31.7% blue/66.3% red, 121 ± 7 µmol m^−2^·s^−1^) LB (14.8% blue/81.3% red, 121 ± 7 µmol m^−2^·s^−1^)14-h photoperiod	Growth	HB light decreased the contents of progoitrin. BL/RL light ratios slightly affect GLSs content.	[169]
Broccoli (*Brassica oleracea* var. *italica*)	(A) FL (B) BL/RL (5% 447 nm/95% 627 nm) (C) BL/RL/GL (5%/85%/10% 530 nm) (D) BL/RL (20%/80%) (E) BL/RL/GL (20%/70%/10%). 250 µmol m^−2^·s^−1^ ; 16-h photoperiod	Growth	D > C > B > E > A increased the level of aliphatic, indoles, and total GLS.	[175]
Broccoli (*Brassica oleracea* var. *italica*)	BL + RL (12% 470 nm/88% 627 nm; 350 µmol m^−2^·s^−1^); BL (470 nm; 41 ± 2 µmol m^−2^·s^−1^). 24-h photoperiod	Growth	BL increased the level of glucoraphenin, epiprogoitrin, and aliphatic GLSs, while indole GLSs were not impacted by the LED lighting treatment.	[176]
*Cardamine fauriei*	RL (660 nm), BL (445 nm), GL (520 nm) RL + BL (1:1), 75 µmol m^−2^·s^−1^; 16-h photoperiod	Growth	BL increased accumulation of aliphatic GSLs and decreased accumulation of indolic GSL	[177]
Choy sum (*Brassica rapa* subsp. *chinensis* var. *parachinensis*)	RB: 160 μmol m^−2^·s^−1^ and 80 μmol m^−2^·s^−1^; WL: 160 W and 80 W; BL (450 nm) RL (660 nm). 12-h photoperiod	Growth	No significant difference in the total GSL content was observed in response to the various LED light treatments within the same growth stage. The changes were more pronounced across growth stages.	[178]
(*Brassica napus L.* ssp*. rapifera* Metzg)	RL (660 nm), FR (740 nm), RL/FRL (1:1), BL (460 nm); 24-h photoperiod;10 μmol m^−2^·s^−1^.These treatments were given in combination with temperature regimes of either constant 15 °C (100 day) or 15 °C (65 day) followed by 9 °C (66–100 day)	Growth	Total GLS concentrations were not different among treatments, progoitrin was present in highest concentration under LEDs containing far-red light, and in lower concentration at 9 °C compared to 15 °C	[179]

## Supplementary Materials

The following are available online at https://www.mdpi.com/2076-3921/10/1/42/s1, Figure S1: Schematic representation of (A) the L-galactose pathway of vitamin C (L-ascorbate) biosynthesis and (B) the Ascorbate-glutathione cycle. Figure S2. Schematic representation of the polyphenol biosynthetic pathway. Figure S3. Schematic representation of the chlorophyll biosynthetic pathway. Figure S4. Schematic representation of the carotenoids biosynthetic pathway. Figure S5. Schematic representation of the glucosinolates biosynthetic pathway.
