# Supplementary material for: Challenges and Opportunities of Light-Emitting Diode (LED) as Key to Modulate Antioxidant Compounds in Plants. A Review
Source: Antioxidants (Basel). 2020 Dec 31;10(1):42. doi: 10.3390/antiox10010042 (PMC7824119; doi:10.3390/antiox10010042)
Supplement: Supplementary file 1 [file antioxidants-10-00042-s001.pdf]

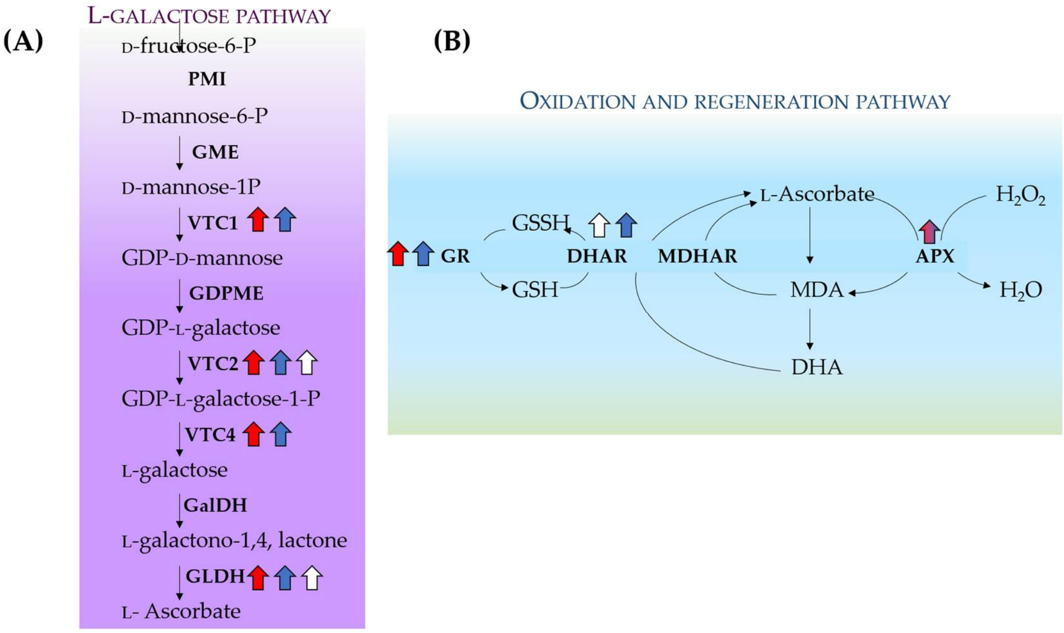

Figure S1. Schematic representation of **A**) the L-galactose pathway of vitamin C (L-ascorbate) biosynthesis and **B**) the Ascorbate-glutathione cycle. Arrows indicate up- or down-regulation by blue ( ), red ( ), or white ( ) LEDs. Adapted from the MetaCyc database (https://biocyc.org/). Phosphomannose isomerase (PMI), GDP-D-mannose 3′,5′-epimerase (GME), GDP-D-mannose pyrophosphorylase (VTC1), GDP-mannose 3’,5’-epimerase (GDPME), GDP-L-galactose phosphorylase (VTC2), l-galactose-1-phosphate phosphatase (VTC4), l-galactose dehydrogenase (GalDH), l-galactono-1,4-lactone dehydrogenase (GLDH), ascorbate peroxidase (APX), monodehydroascorbate reductase (MDHAR), dehydroascorbate reductase (DHAR), glutathione reductase (GR), monodehydroascorbate (MDA), dehydroascorbate (DHA), reduced glutathione (GSH), oxidized glutathione (GSSH).

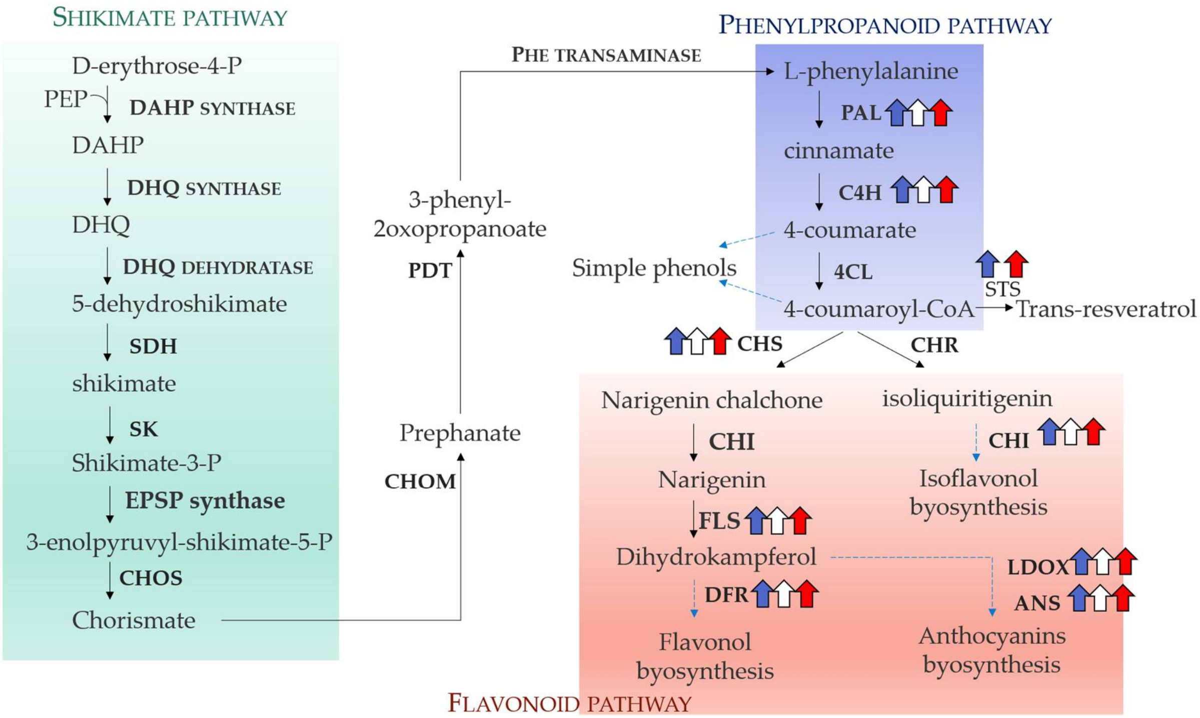

Figure S2. Schematic representation of the polyphenol biosynthetic pathway. Arrows indicate up- or down-regulation by blue ( ), red ( ), or white ( ) LEDs. Adapted from the MetaCyc database (https://biocyc.org/). Phosphoenolpyruvate (PEP), 3-deoxy-D-arabino-heptulosonate-7-phosphate (DHAP), 3-dehydroquinate (DHQ), 5-dehydroshikimate dehydrogenase (SDH), shikimate kinase (SK), 3-enolpyruvyl-shikimate-5-phospate synthase (EPSP synthase), chorismate synthase (CHOS), chorismite mutase (CHOM), prephenate dehydratase (PDT), phenylalanine (PHE), phenylalanine ammonia lyase

(PAL), cinnamate 4-hydroxylase (C4H), 4-coumarate coenzyme A ligase (C4H), chalcone synthase (CHS), chalcone isomerase (CHI), flavonol synthase (FLS), leucoanthocyanidin dioxygenase (LDOX), dihydroflavonol 4-reductase (DFR), Anthocyanidin synthase (ANS), and stilbene synthase (STS).

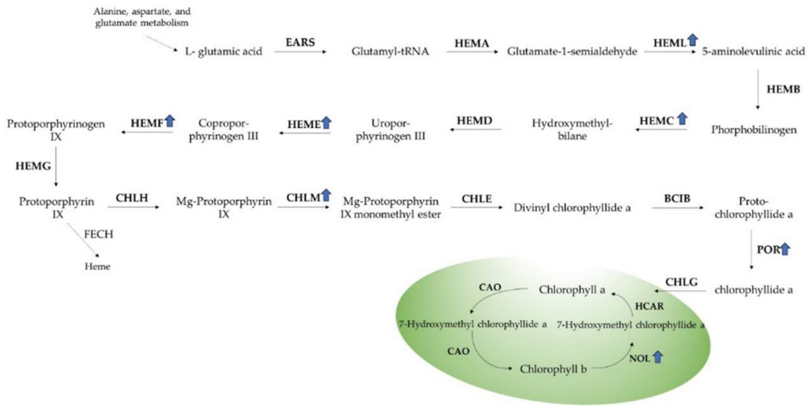

Figure S3. Schematic representation of the chlorophyll biosynthetic pathway. Arrows indicate up- or down-regulation by blue ( ), red ( ), or white ( ) LEDs. Adapted from the MetaCyc database (https://biocyc.org/). Glutamyl-tRNA synthetase (EARS), glutamyl-tRNA reductase (HEMA), glutamate-1-semialdehyde 2,1-aminotransferase (HEML), 5-aminolevulinate dehydrogenase (HEMB), porphobilinogen deaminase (HEMC), uroporphyrinogen III synthase (HEMD), uroporphyrinogen III decarboxylase (HEME), coproporphyrinogen III oxidase (HEMF), protoporphyrinogen oxidase (HEMG), Mg-chelatase (CHLH), Mg-protoporphyrin IX methyltransferase (CHLM), magnesium-protoporphyrin IX monomethyl ester cyclase (CHLE), 3,8- divinyl protochlorophyllide a 8-vinyl-reductase (ferredoxin) (BCIB), Protochlorophyllide reductase (POR), chlorophyll a synthase (CHLG), protoporphyrin/coproporphyrin ferro chelatase (FECH),chlorophyllide a oxygenase (CAO), chlorophyll b reductase (NOL), 7-Hydroxymethyl chlorophyll a reductase (HCAR).

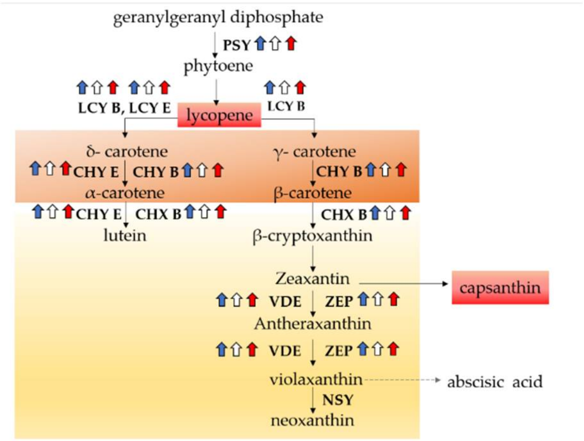

Figure S4. Schematic representation of the carotenoids biosynthetic pathway. Arrows indicate up- or down-regulation by blue ( ), red ( ), or white ( ) LEDs. Adapted from the MetaCyc database (https://biocyc.org/). Phytoene synthase (PSY), lycopene β-ring cyclase (LCYB), lycopene ε-ring cyclase (LCYE), carotenoid β-ring hydroxylase, (CHYB), carotenoid ε-ring hydroxylase (CHYE), zeaxanthin epoxidase (ZEP), violaxanthin de-epoxidase (VDE), neoxanthin synthase (NSY).

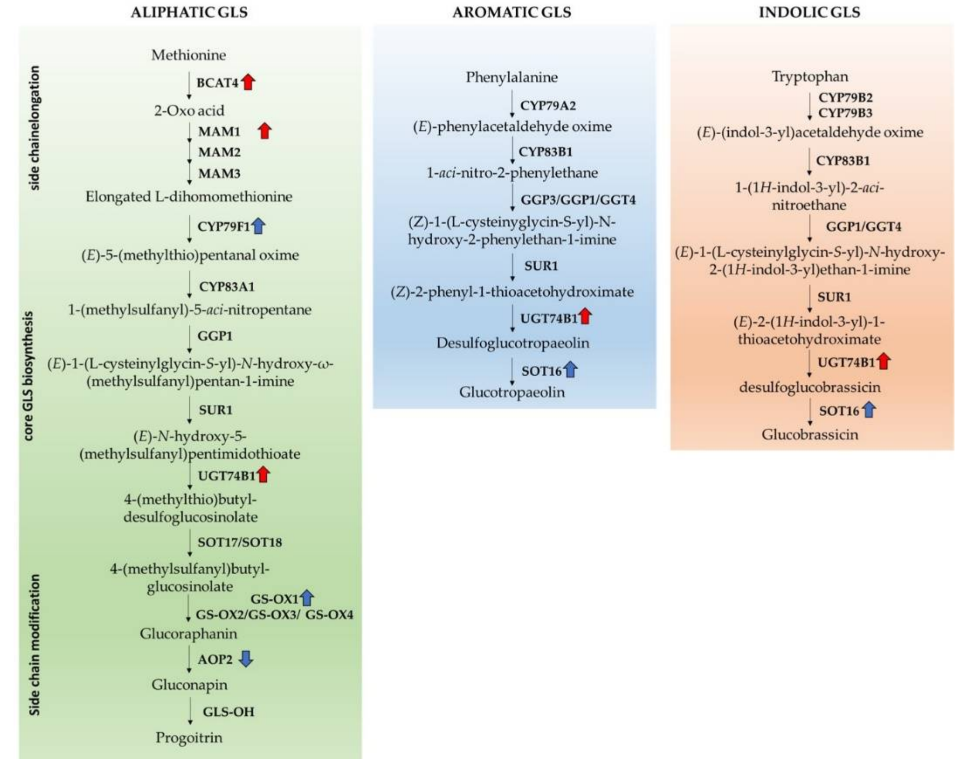

Figure S5. Schematic representation of the glucosinolates biosynthetic pathway. Arrows indicate up- or down-regulation by blue (⇧), red (⇧), or white (⇧) LEDs. Adapted from MetaCyc database (https://biocyc.org/). branched-chain aminotransferase (BCAT), methylthioalkylmalate synthase (MAM1), homomethionine monooxygenase (CYP79F1/F2), methylthiobutanaldoxime monooxygenase (CYP83A1), methylthiobutylhydroximoyl-glutathione gamma-glutamyl peptidase (GGP1), (E)-1-(glycylcystein-S-yl)-4-(methylthio)-butylhydroximate C-S lyase (SUR1), N-hydroxythioamide S-β-glucosyltransferase (UGT74B1), 3-methylthiopropyl-desulfoglucosinolate sulfotransferase (SOT), omega-(methylthio)alkyl-glucosinolate S-oxygenase (GS-OX), glutathione-S conjugate gamma-glutamyl hydrolase (GGT4), UDP-glucose: 4-methylthiobutylhydroximate S-glucosyltransferase (UGT74B1), 3-(methylsulfinyl)propyl-glucosinolate dioxygenase (AOP2), 3-butenylglucosinolate 2-hydroxylase (GLS-OH).
